# On the origin of PRDM9-guided recombination hotspots

**DOI:** 10.1073/pnas.2535682123

**Published:** 2026-06-25

**Authors:** Francisco Úbeda, Reinhard Bürger, Frederic Fyon

**Affiliations:** ^a^ Department of Biology, Royal Holloway University of London, Egham TW20 0EX, United Kingdom; ^b^ Faculty of Mathematics, University of Vienna, Vienna 1090, Austria

**Keywords:** gene conversion, mutation, recombination, population genetics, mathematical biology

## Abstract

Meiotic recombination is highly conserved across vertebrates and plays an essential role in ensuring chromosomal segmentation and generating genomic variation. However, species employ two strikingly different mechanisms to guide recombination. In most mammals, recombination hotspot locations are determined by the protein PRDM9, which binds specific DNA sequence motifs. These motifs are rapidly eroded by biased gene conversion, rendering this mechanism self-destructive and evolutionarily transient. In contrast, birds initiate recombination at open chromatin regions independently of sequence motifs, producing self-preserving and evolutionarily stable recombination hotspots. Some species use both types of hotspots simultaneously, raising the unresolved question of why PRDM9-guided hotspots persist alongside a robust alternative. Here, we address this problem using a population genetic model that explicitly considers competition between PRDM9-guided and non-PRDM9-guided recombination hotspots. We show that non-PRDM9-guided hotspots are generally favored because, lacking sequence specificity, they generate more crossovers required for proper chromosome segregation. However, PRDM9-guided hotspots can overcome this disadvantage when simultaneous binding of both homologous chromosomes (symmetric binding) is more likely to resolve as crossovers than binding of a single homolog (asymmetric binding). Although PRDM9 reduces overall crossover rate, its sequence specificity increases the probability of symmetric binding within hotspots, creating a trade-off that can favor PRDM9 under specific selection regimes. Our model predicts that PRDM9-dependent species are particularly sensitive to disruptions of symmetric binding, whereas species lacking PRDM9 are more vulnerable to complete crossover failure. Intermediate regimes allow stable coexistence of both hotspot types.

## Significance

Across vertebrates, meiotic recombination is guided either by the protein PRDM9, which targets DNA sequences that are gradually destroyed, or by non-PRDM9 mechanisms that act in open chromatin without sequence specificity. Why a self-destructive mechanism evolves alongside a durable alternative remains unresolved. Using a population genetic model, we show that PRDM9-guided hotspots are generally disfavored because they generate fewer binding events and thus fewer crossovers needed to produce viable gametes. However, they can be favored when they promote simultaneous binding of both homologous chromosomes (symmetric binding), which more often produces crossovers. The evolutionary fate of PRDM9 thus reflects a trade-off between reduced binding and enhanced symmetric binding, explaining the diversity and evolutionary turnover of recombination mechanisms observed across vertebrates today.

Meiotic recombination—the process by which genes are reshuffled—occupies a central place in genetics (1, 2). It plays a fundamental role in generating genetic diversity, promoting adaptation and speciation, and preventing genetic conflict (3–6). In most eukaryotic species, recombination is not uniformly distributed across the genome; instead, it is concentrated in small regions where the probability of recombination is several orders of magnitude greater than the genomic average. These regions are known as recombination hotspots (7–10).

**Fig. 1.**
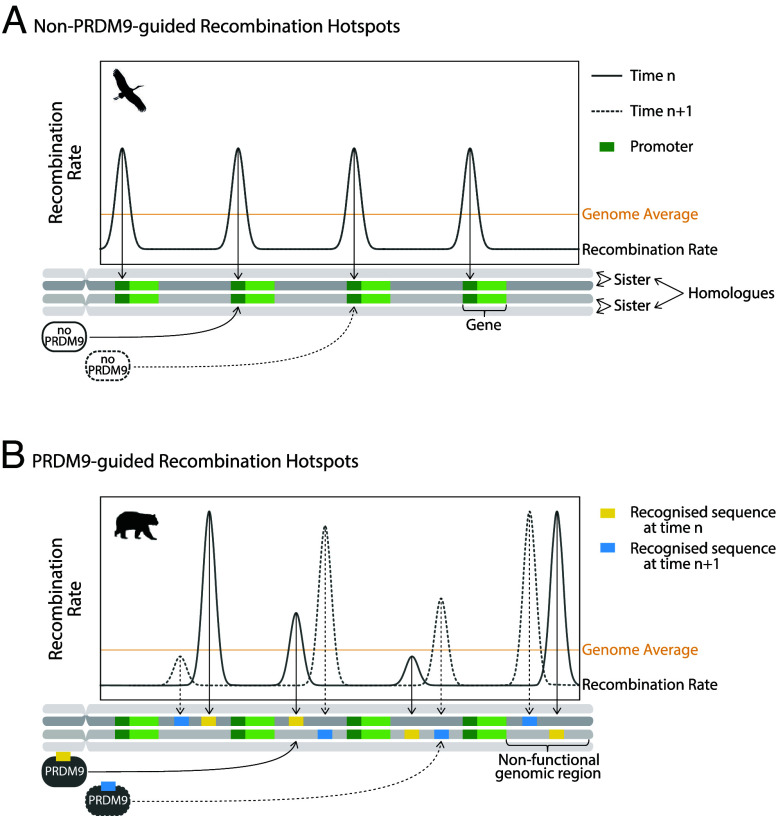
Types of hotspots. (*A*) summarizes the characteristics of non-PRDM9 recombination hotspots: i. recombination machinery does not recognize specific sequences to initiate recombination; ii. recombination takes place in promoter regions; iii. the rate of recombination remains constant over time (self-preserving hotspots); iv. the location of hotspots in the genome does not change over evolutionary time. (*B*) summarizes the characteristics of PRDM9 recombination hotspots: i. PRDM9 recognizes and binds specific sequences recruiting the recombination machinery to initiate recombination in those sequences; ii. recombination takes place in nonfunctional genomic regions; iii. the rate of recombination decreases over time (self-destructive hotspots); iv. the location of hotspots changes rapidly over evolutionary time.

In most mammals, including humans, the gene PRDM9 encodes a protein that binds specific DNA sequence motifs (Fig. 1*B*) (10–13). PRDM9 proteins recruit the machinery that initiates recombination by inducing a double-strand break (7, 14, 15). PRDM9-guided recombination hotspots are therefore located in genomic regions enriched for the motif recognized by the PRDM9 protein (16). During recombination, the bound and broken sequence is converted into its homologous unbound sequence (7, 10, 17, 18). In other words, the sequence that enables recombination (the hot allele) is converted into a sequence that prevents recombination (the cold allele) (7, 10, 17, 18). Biased gene conversion in favor of the cold allele leads to a gradual decay of recombination activity at PRDM9 hotspots over evolutionary time (19–22) (Fig. 1*B*). In this sense, PRDM9 recombination hotspots are self-destructive.

In contrast, in most ray-finned fishes and all birds, the PRDM9 gene is either absent or nonfunctional (Fig. 1*A*). In these species, the recombination machinery binds directly to DNA in gene promoter regions, most likely because their open chromatin structure facilitates the binding of proteins that induce double-strand breaks (13, 23). Non-PRDM9-guided recombination hotspots are therefore found in genomic regions that are more accessible to the recombination machinery. Because the machinery does not bind a specific DNA sequence, gene conversion is unbiased, and non-PRDM9 recombination hotspots do not decay over evolutionary time (23, 24) (Fig. 1*A*). In this sense, non-PRDM9 recombination hotspots are self-preserving.

The difference in sequence specificity between these two types of hotspots results in two dramatically different recombination landscapes (Fig. 1). In organisms with PRDM9 recombination hotspots, selection favors PRDM9 alleles that target more abundant sequences whereas the original target motif becomes less abundant due to biased gene conversion. This selection pressure arises because the absence of crossovers leads to more frequent abnormal meiotic divisions (12, 25, 26). As a result, individual recombination hotspots disappear (die) as hot alleles are lost, and selection then favors the formation (birth) of new hotspots through the fixation of mutant PRDM9 alleles that bind novel motifs (25, 26). Consequently, the recombination landscape in species with PRDM9 hotspots is characterized by changes in both recombination rate (intensity) and spatial distribution (position) over time. This pattern is often referred to as a variable landscape (27–30) (Fig. 1). Coevolution between PRDM9 and its targets preserves the average number of recombination hotspots—and the average recombination rate—across the genome (26). In contrast, with non-PRDM9 recombination hotspots, the lack of specificity for any target sequence results in individual hotspots that never disappear. The recombination landscape in species with non-PRDM9 hotspots is therefore characterized by constant hotspot intensity and position over time, often referred to as a stable landscape (23, 24) (Fig. 1).

**Fig. 2.**
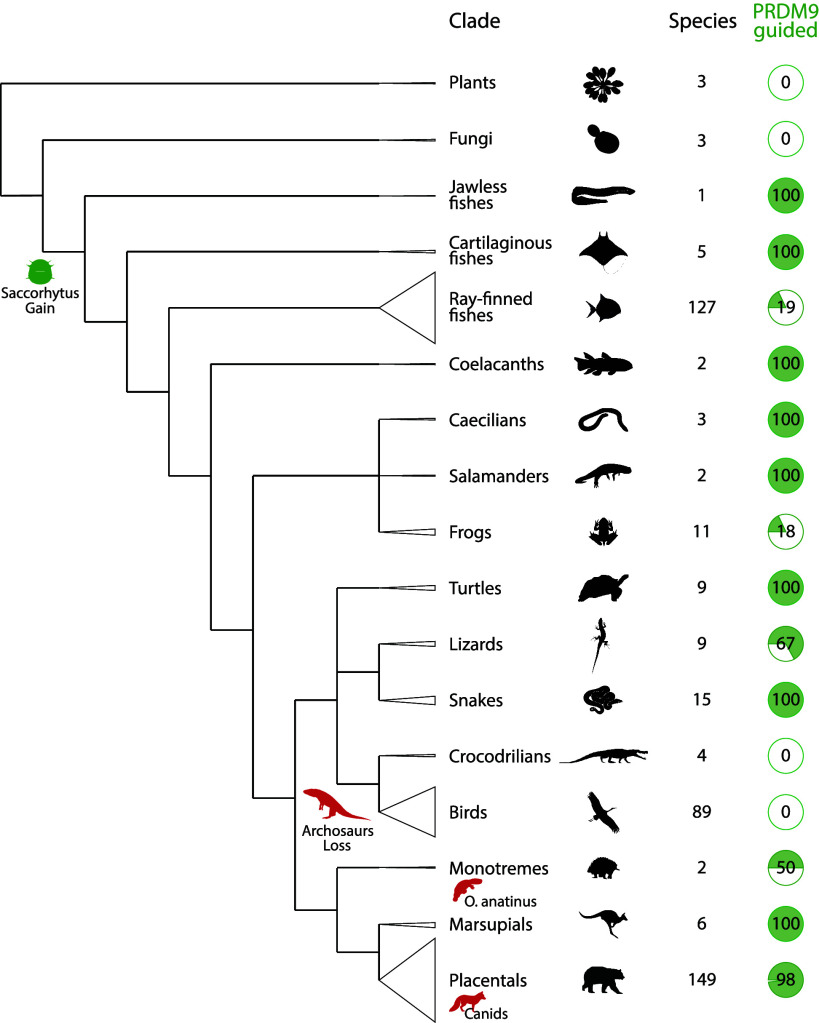
Phylogenetic distribution of PRDM9 recombination hotspots. For each clade we present the number of species that have been researched and the percentage of them where there is evidence of PRDM9 recombination hotspots (adapted from ref. 13 with additional data from refs. 24 and 31–33). We use green to represent a gain of PRDM9 recombination hotspots in the ancestor of vertebrates, and red to represent some examples of the multiple losses of PRDM9 recombination hotspots, for example, in the ancestor of birds and crocodiles, the ancestor of canids, or the ancestor of platypus.

Evidence suggests that multiple transitions between these two types of recombination hotspots have occurred throughout evolutionary time (Fig. 2). Non-PRDM9 hotspots are observed in plants (31, 33, 34) and yeast (24). PRDM9 hotspots are observed in vertebrates (13), with at least thirteen independent losses in lineages including frogs, crocodiles, and birds, as well as several losses in ray-finned fish clades (13, 35) (Fig. 2). The split between plant and fungal lineages, and vertebrates predates the origin of vertebrates, suggesting that the non-PRDM9 mechanism is the ancestral character state. In addition, the default recombination mechanism in some mammals, including humans, relies on non-PRDM9 hotspots, with PRDM9 hotspots acting as a redundant mechanism (16, 36–38). For example, knockout of PRDM9 in mice does not eliminate recombination but instead results in a redistribution of recombination hotspots to open chromatin regions (16, 37). Taken together, these observations suggest that non-PRDM9 hotspots are ancestral, that PRDM9 hotspots evolved prior to the common ancestor of vertebrates, and that they have been repeatedly lost since (31, 32, 39, 40) (Fig. 2). Independently of ancestry, the distribution of both hotspot types across clades provides evidence for multiple transitions between mechanisms (Fig. 2). Remarkably, the presence of one hotspot type does not always exclude the other: Recent work shows that some species of mammals and snakes simultaneously use both types of recombination hotspots (40, 41).

What drives the evolution of one type of recombination hotspot in the presence of the other remains unknown. What are the evolutionary origins of each type? Which transitions are favored by natural selection? How can natural selection favor the coexistence of both types of hotspots? Why does selection favor different recombination mechanisms in different clades? These questions remain unanswered, and there is currently no model describing the competition between the two hotspot types. In this research, we address these questions.

To determine which type of hotspot is favored by natural selection, we develop a population genetic model consisting of a targeting locus that produces a protein initiating recombination at a target locus, and a modifier locus that alters the specificity of the interaction between the targeting and target loci. We use this model to explore the evolution of specificity encoded by the modifier locus under coevolution between targeting and target loci. Lack of specificity represents non-PRDM9 recombination hotspots, whereas specificity represents PRDM9 recombination hotspots. We examine whether rare PRDM9 hotspots can invade populations initially possessing only non-PRDM9 hotspots, and conversely, whether rare non-PRDM9 hotspots can invade populations with only PRDM9 hotspots. This analysis sheds light on the evolutionary stability of each recombination mechanism. Ultimately, our results provide insight into the gains and losses of PRDM9 hotspots and the distribution of recombination hotspot types across clades.

## Methods

### Model.

In this section we introduce the parameters needed to formulate our model and present the equations for the change in gamete frequencies across generations. A detailed derivation can be found in *SI Appendix*.

We assume an infinite population of diploid individuals that reproduce sexually in discrete generations. We model the coevolution of three loci: the modifier locus M, the targeting locus A, and the target locus B. Locus A carries alleles $A_{k}$ (where k∈{1,2}), each encoding proteins that attempt to bind a DNA base-pair motif at locus B. Locus B carries alleles $B_{m}$ (where m∈{1,2}), each corresponding to sequence motifs for which the protein encoded by locus A may show differential binding affinity. Locus M carries alleles $M_{i}$ (where i∈{0,1}) that determine whether the protein synthesized by locus A binds all motifs segregating at locus B with equal probability—thus showing no specificity—or binds one motif with higher probability than the other—thus inducing specificity.

We model two alleles at each locus for reasons of mathematical tractability. This simplification results in the same hotspot oscillating between hot and cold states, where the population is in a hot (cold) state if the target allele $B_{k}$ is predominantly paired with the matching targeting allele $A_{k}$ (mismatching $A_{m}$). In reality, it will be a hotspot becoming hot in the first place and cold afterward followed by the same process at another hotspot, and so on. We have shown elsewhere and discuss in *SI Appendix* that whether it is the same or a different target site does not have an impact on the coevolution of targeting and target sites (25, 26, 42). As such our diallelic model benefits from mathematical tractability with little loss of generality.

Let $x_{i,k,m}$ denote the frequency of gamete $M_{i}A_{k}B_{m}$ in the initial pool, where $\sum_{i,k,m}x_{i,k,m}=1$. We model the change in frequency of gametes in a life-cycle consisting of five steps (cf. *SI Appendix*, Fig. S1).

#### Random mating.

Random union of haploid gametes results in a population of diploid individuals.

#### Symmetric and asymmetric binding.

Our model closely follows the molecular mechanisms underlying PRDM9-guided recombination hotspots. In species where PRDM9 is present and functional, a match between the zinc-finger domain of the PRDM9 protein and a DNA motif results in binding with some probability, whereas a mismatch results in no binding. In species where the gene PRDM9 is either absent or nonfunctional, the recombination machinery binds to open chromatin regions with some probability, independently of the underlying DNA sequence motif. In our model, homozygotes for the specific allele $M_{1}$ synthesize proteins that bind a particular motif with probability b (0<b≤1) when subscripts match, but do not bind when subscripts do not match. These genotypes therefore behave analogously to functional PRDM9. Homozygotes for the unspecific allele $M_{0}$, in contrast, synthesize proteins that bind any motif with the same probability β (0<β≤1), independently of whether subscripts match. These genotypes thus behave as if PRDM9 were absent or nonfunctional. For simplicity, and given the lack of empirical evidence favoring any alternative, our model assumes no dominance between alleles at the modifier locus.

Our model assumes that the genotype-averaged total binding probabilities, i.e., the probabilities that binding occurs, are identical for homozygous specific ($M_{1}M_{1}$) and homozygous unspecific ($M_{0}M_{0}$) genotypes. By genotype-averaged total binding probabilities we mean binding probabilities averaged over all possible two-locus genotypes $\frac{A_{k}B_{m}}{A_{l}B_{n}}$, obtained by integrating over all two-locus zygotic frequencies (i.e., $x_{0,k,m}x_{0,l,n}$ versus $x_{1,k,m}x_{1,l,n}$). Straightforward calculations show that the genotype-averaged total binding probabilities are equal for the $M_{1}M_{1}$ and $M_{0}M_{0}$ backgrounds (and hence for $M_{0}M_{1}$) if and only if the unspecific binding probability satisfies $\beta =\frac{1}{2}b$. We explicitly impose this condition. The rationale is simple: Because we are interested in competition between specific and unspecific phenotypes, and because fitness depends on the probability of crossovers—which require binding—assigning unequal genotype-averaged binding probabilities would automatically confer a direct fitness advantage to the phenotype with the higher binding probability. Numerical iterations confirm this intuition.

Despite equal genotype-averaged binding probabilities, the specific and unspecific phenotypes differ in their binding probabilities for most individual genetic backgrounds. Under our assumptions, the unspecific phenotype exhibits a constant binding probability of $\frac{1}{2}b$ regardless of the targeting–target genotype. The specific phenotype, by contrast, binds with probability b in hot genetic backgrounds ($x_{1,k,k}\approx 1$) and with probability 0 in cold genetic backgrounds ($x_{1,k,m}\approx 0$ for m≠k). Thus, in hot backgrounds the specific phenotype is more likely to bind than the unspecific phenotype ($b>\frac{1}{2}b$), whereas in cold backgrounds it is less likely to bind ($0<\frac{1}{2}b$). Binding probabilities for heterozygous genotypes are provided in *SI Appendix*.

Finally, the model assumes that proteins attempt to bind both motifs at the target locus. The possible outcomes are that proteins bind both homologous targets (symmetric binding), bind only one target (asymmetric binding), or fail to bind either target. This assumption generalizes previous coevolutionary models (25, 26, 43) and allows us to consider the possibility that PRDM9 and non-PRDM9-guided mechanisms affect symmetric and asymmetric binding differently. Such differences have been highlighted by recent empirical and theoretical work on PRDM9 (22, 39, 44, 45). For a genotype $\frac{M_{i}A_{k}B_{m}}{M_{j}A_{l}B_{n}}$, the probabilities of symmetric, asymmetric, and no binding are denoted $B_{ij,kl,mn}^{s}$, $B_{ij,kl,mn}^{a}$, and $B_{ij,kl,mn}^{0}$. Importantly, these binding probabilities are not imposed as explicit assumptions but arise naturally from applying standard probabilistic rules to interactions between matching and mismatching alleles at the targeting and target loci (*SI Appendix*).

#### Double-strand break, conversion, and selection.

Binding induces a double-strand break that initiates recombination. In cases of asymmetric binding, only one sequence is bound, and thus only that motif can experience a double-strand break. In contrast, during symmetric binding both homologous motifs are bound, and either may undergo a double-strand break. In this case, we assume that the two motifs are equally likely to experience the break.

The broken chromatid can be repaired using its homologous chromatid as a template (7, 46). When recombination is guided by PRDM9-proteins, gene conversion is biased because the proteins preferentially induce breaks at matching motifs (hot alleles) rather than mismatching motifs (cold alleles). As a consequence, hot alleles are converted into cold alleles with probability c, where 0<c≤1 (7, 10, 17, 18). The overtransmission of cold alleles explains why PRDM9 recombination hotspots are self-destructive (7, 19). In contrast, when recombination is guided by non-PRDM9 proteins, gene conversion is unbiased because unspecific proteins are equally likely to break either motif. In this case, hot and cold alleles do not exist, and any allele is converted into its homolog with equal probability. The equal transmission of alleles explains why non-PRDM9-guided recombination hotspots are self-preserving.

Consistent with previous theoretical work and empirical observations, we assume that a double-strand break at the target locus leads either to proper alignment of homologous chromatids or to crossover formation, resulting in correct chromosome segregation into gametes (19, 20, 26, 47–51). By contrast, the absence of a double-strand break leads to defective chromosome segregation. Then gametes are nonviable (e.g., due to aneuploidy) with probability f, where 0<f≤1 (19, 20, 26, 47–51).

Recent studies suggest that the type of binding leading to a double-strand break also affects gamete viability. In particular, symmetric binding appears more likely to resolve as crossover and result in proper chromosomal segregation than asymmetric binding (22, 44, 45, 52). Therefore we introduce the parameters $\rho_{s}$ and $\rho_{a}$ which are the probabilities that symmetric and asymmetric binding resolve as crossover, respectively. We assume $0<\rho_{a}\leq \rho_{s}\leq 1$. Hence, the expected fitness of individuals experiencing symmetric, asymmetric, or no binding is $F^{s}=1-f\left(1-\rho_{s}\right)$, $F^{a}=1-f\left(1-\rho_{a}\right)$, and $F^{0}=1-f$, resp., where $0<F^{0}\leq F^{a}\leq F^{s}\leq 1$.

#### Recombination.

Recombination between alleles at the three loci occurs either before or after gene conversion (with which it formally commutes), but before selection, which takes place at the end of meiosis. For simplicity, we assume independent assortment of alleles at all loci. Then the frequency of the gamete $M_{i}A_{k}B_{m}$ after recombination, conversion, and selection is given by

[1] $$\begin{array}{cc} \overset{¯}{w}x_{i,k,m}^{\left(rcs\right)}= & \sum_{j,l,n}\left[\left(F^{0}B_{ij,kl,mn}^{0}+F^{a}\left(1-\frac{1}{2}c\right)B_{ij,kl,mn}^{a}\right.\right.\\ & \left.\left.+F^{s}B_{ij,kl,mn}^{s}\right)+\frac{1}{2}cF^{a}B_{ij,kl,nm}^{a*}\right]{\left(x_{i,k,m}x_{j,l,n}\right)}^{\left(r\right)}. \end{array}$$

Here, ${\left(x_{i,k,m}x_{j,l,n}\right)}^{\left(r\right)}=\frac{1}{4}(x_{i,k,m}x_{j,l,n}+x_{i,k,n}x_{j,l,m}+x_{i,l,n}x_{j,k,m}+x_{i,l,m}x_{j,k,n})$ is the frequency of the genotype $\frac{M_{i}A_{k}B_{m}}{M_{j}A_{l}B_{n}}$ after free recombination among the three loci,

[2] $$\begin{array}{cc} \overset{¯}{w}= & \sum_{i,k,m}\sum_{j,l,n}(F^{0}B_{ij,kl,mn}^{0}+F^{a}B_{ij,kl,mn}^{a}+F^{s}B_{ij,kl,mn}^{s})\\ & \times x_{i,k,m}x_{j,l,n} \end{array}$$

is the population mean fitness, and $B_{ij,kl,mn}^{a*}$ is the probability that an asymmetric double-strand break occurs in motif m (see *SI Appendix* for details).

#### Mutation.

Following the segregation of haplotypes into viable gametes we model recurrent mutation. We assume alleles at locus A mutate from $A_{k}$ to $A_{l}$ with probability $\mu_{A,kl}=\mu_{A}$, where k≠l, and do not mutate with probability $\mu_{A,kk}=1-\mu_{A}$, where $0<\mu_{A}<1$. Similarly, we assume alleles at locus B mutate from $B_{k}$ to $B_{l}$ with probability $\mu_{B,kl}=\mu_{B}$, where k≠l, and do not mutate with probability $\mu_{B,kk}=1-\mu_{B}$, where $0<\mu_{B}<1$. We do not allow recurrent mutation at the modifier locus, but we study the evolution of mutant alleles at the modifier locus as central part of our investigation.

The frequency of gamete $M_{i}A_{k}B_{m}$ in the next generation is

[3] $$\begin{array}{c} x_{i,k,m}^{\prime }=\sum_{l,n}\mu_{A,lk}\mu_{B,nm}x_{i,l,n}^{\left(rcs\right)}\;. \end{array}$$

Mutation brings us back to the beginning of our life-cycle.

Rather than working directly with gamete frequencies, we find it more informative to describe the dynamics in terms of allele frequencies and linkage disequilibria. Let $m=\sum_{k,m}x_{1,k,m}$ denote the frequency of the specificity allele $M_{1}$, $p=\sum_{i,m}x_{i,1,m}$ the frequency of the targeting allele $A_{1}$, and $q=\sum_{i,k}x_{i,k,1}$ the frequency of the target allele $B_{1}$. Let $D_{\mathrm{MA}}$, $D_{\mathrm{MB}}$, and $D_{\mathrm{AB}}$ denote the pairwise linkage disequilibria measuring associations between the indicated loci. The exact recursions also require the three-way linkage disequilibrium $D_{\mathrm{MAB}}$ among all three loci. Precise definitions of these quantities are provided in *SI Appendix*. Importantly, $D_{\mathrm{AB}}$ is negative if there is an association between mismatching alleles at the targeting and target loci, i.e., if $A_{1}$ ($A_{2}$) is associated with $B_{2}$ ($B_{1}$).

The full recursion equations for allele frequencies and linkage disequilibria are algebraically complex (*SI Appendix*, Supplementary *Mathematica* notebook) (53). Representative examples of the evolutionary dynamics are shown in *SI Appendix*, Figs. S2–S6. Here, we focus on the change in frequency m of the specific modifier allele $M_{1}$. Extensive numerical iteration of the recursion Eq. **3** shows that the single-generation change in modifier frequency, $\overset{¯}{w}\Delta m$, is well approximated by

[4] $$\begin{array}{cc} \overset{¯}{w}\Delta m & \approx m\left(1-m\right)\frac{1}{4}bf[\rho_{a}\left(1-2p\right)\left(1-2q\right)\\ & +2\rho_{a}D_{\mathrm{AB}}+S_{\rho }], \end{array}$$

where $S_{\rho }=b\left(\rho_{s}-\rho_{a}\right)[\left(1-2p\right)\left(1-2q\right)+\frac{1}{2}({\left(1-p\right)}^{2}+p^{2}){\left(1-2q\right)}^{2}+4(\left(1-p\right)\left(1-q\right)+pq)D_{\mathrm{AB}}]$ captures the additional contribution arising when symmetric binding confers a fitness advantage over asymmetric binding. We discuss the evolutionary insights provided by this fundamental equation when presenting our main results.

### Numerical Analysis.

In general, because some of the dynamics we consider are oscillatory, initial increases in the frequency of rare alleles—i.e., invasion conditions—do not necessarily predict the probability that a mutant allele will ultimately replace the resident allele. Rather than analyzing invasion conditions in detail, we therefore rely on numerical analysis to characterize the long-term fate of mutant alleles. In all cases, this is done by forward iteration of the deterministic recursion Eq. **3** for at least 50,000 generations. Our aim is to explore the evolution of PRDM9 versus non-PRDM9 recombination hotspots when they differ only in their underlying mechanism.

We first perform a set of simulations to study the frequency dynamics of a mutant specific allele $M_{1}$ in populations where the unspecific allele $M_{0}$ is fixed. In these simulations, we assess whether the mutant specific modifier can replace the resident unspecific modifier over evolutionary time. In particular, we examine how fertility reductions associated with the absence of binding (f), asymmetric binding ($f-f\rho_{a}$), and symmetric binding ($f-f\rho_{s}$) influence the probability of replacement. We assume that the initial frequency of the rare specific modifier is m=0.01. In populations where the unspecific modifier $M_{0}$ is fixed (its frequency is 1−m≈1), any pair of allele frequencies at the targeting and target loci constitutes a selectively neutral state (*SI Appendix*). Accordingly, we allow the initial frequencies at these loci to take any pair of values $\left(p^{0},q^{0}\right)$. For each parameter combination, we sample 500 initial conditions with $p^{0}$ and $q^{0}$ drawn uniformly from between 0 and 1. In all cases, we assume that initially there is no linkage disequilibrium among the three loci, i.e. $D_{\mathrm{MA}}^{0}=D_{\mathrm{MB}}^{0}=D_{\mathrm{AB}}^{0}=D_{\mathrm{MAB}}^{0}=0$. If m>0.9999 at the end of the set iterations, the specific allele $M_{1}$ is considered fixed; if m<0.0001, the unspecific allele $M_{0}$ is considered fixed.

We then carry out a second set of simulations to investigate the frequency dynamics of a mutant unspecific allele $M_{0}$ in populations where the specificity allele $M_{1}$ is fixed. In these simulations, we determine whether the mutant unspecific modifier can replace the resident specific modifier over evolutionary time. As above, we examine how f, $\rho_{a}$, and $\rho_{s}$ influence the probability of replacement. We assume that the initial frequency of the rare unspecific modifier is 1−m=0.01. In populations where the specific modifier $M_{1}$ is fixed (m≈1), different dynamical regimes arise depending on the magnitude of fertility loss due to the absence of binding:

1. When fertility loss is high, $f>f_{\mathrm{cyc}}$ (see *SI Appendix* for the expression of $f_{\mathrm{cyc}}$), there are two internal equilibria near the hot corners of the parameter space. At these equilibria, the targeting locus is nearly fixed for one allele, and the target locus is fixed for the matching allele, namely p,q≈1 or p,q≈0 (26).
2. When fertility loss is low, $f<f_{\mathrm{cyc}}$, there is an internal equilibrium at the center of the parameter space where both alleles at each locus are equally frequent, that is $p=q=\frac{1}{2}$. In addition, a stable limit cycle exists along the edges of the parameter space, along which alleles at the targeting and target loci alternate (26).

Hereafter, we focus on the parameter region $f<f_{\mathrm{cyc}}$, where recombination hotspots alternate between hot and cold states, as this behavior corresponds to empirical observations of rapidly evolving recombination landscapes. Accordingly, we assume that the initial allele frequencies $\left(p^{0},q^{0}\right)$ lie on the limit cycle observed when the specific modifier is fixed. Because no analytical expression for the limit cycle is available (26), we first run simulations from initial conditions within its basin of attraction (26) and record values once the limit cycle has been reached. From this set of $\left(p^{0},q^{0}\right)$ values, we sample 500 initial conditions. As in the previous set of simulations, we assume that linkage disequilibrium is initially absent and apply the same criteria for replacement of the resident allele.

For some parameter combinations and initial conditions, the replacement criteria are not met. Closer examination of these cases reveals that modifier alleles can exhibit persistent oscillatory dynamics. To characterize these outcomes, we run simulations for up to half a million generations. In these cases, rather than applying the replacement criteria, we plot the frequency of the specificity allele over time to describe the long-term behavior.

Following the classical concept of an evolutionarily stable strategy (54), we define a recombination hotspot type (either PRDM9-guided or non-PRDM9) as evolutionarily stable if, when resident, it cannot be replaced by a rare hotspot of the alternative type. Our simulations allow us to determine the evolutionary stability of each hotspot type and reveal five possible dynamical outcomes: four corresponding to different combinations of evolutionary stability and one characterized by oscillatory coexistence. These outcomes are the following:

1. Non-PRDM9-guided hotspots are the only evolutionarily stable state. Rare non-PRDM9 hotspots can replace resident PRDM9 hotspots, whereas rare PRDM9 hotspots cannot replace resident non-PRDM9 hotspots. In this case, we predict that only non-PRDM9 hotspots persist.
2. PRDM9-guided hotspots are the only evolutionarily stable state. Rare non-PRDM9 hotspots cannot replace resident PRDM9 hotspots, whereas rare PRDM9 hotspots can replace resident non-PRDM9 hotspots. In this case, PRDM9-guided hotspots persist.
3. Non-PRDM9-guided and PRDM9-guided hotspots are evolutionarily stable. Neither type can replace the other when rare, leading to no transitions between hotspot types.
4. Neither non-PRDM9-guided nor PRDM9-guided hotspots is evolutionarily stable: 4.1. Both types can replace the other when rare, leading to repeated transitions between hotspot types. 4.2. Both types of hotspots coexist in the population. In this case, both types of hotspot remain active, with their relative usage changing over time but without either type replacing the other.

## Results

### Equal Probabilities of Symmetric and Asymmetric Binding to Resolve as Crossovers:.

$\rho_{s}=\rho_{a}$.

#### Non-PRDM9 resident and PRDM9 mutant:.

**Fig. 3.**
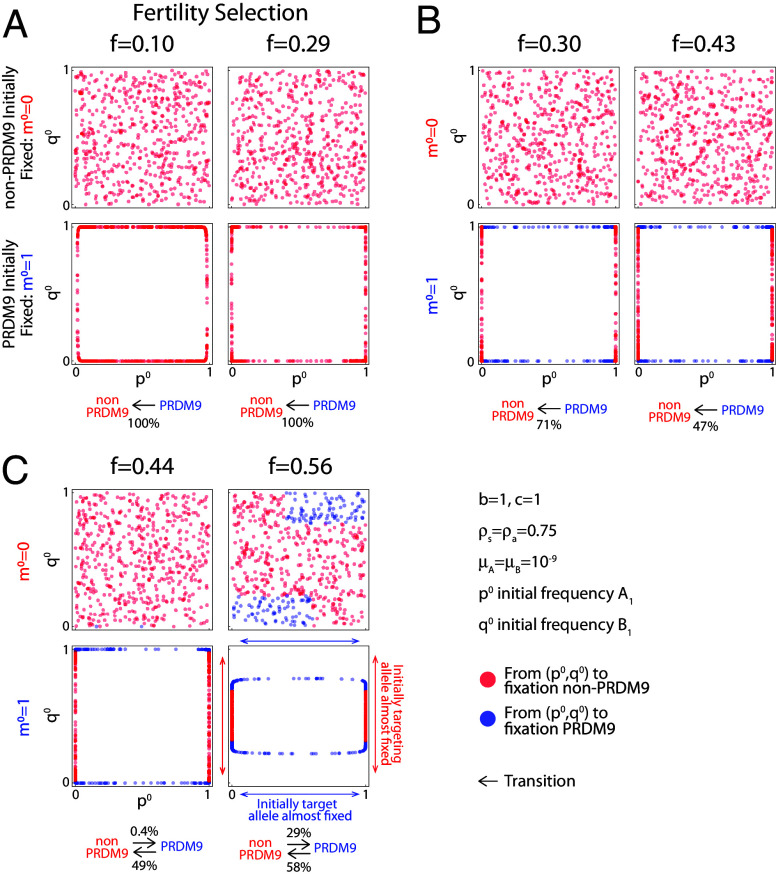
Transitions between types of recombination hotspots when there is no difference between symmetric and asymmetric binding. Each column corresponds to a different fitness cost of no binding (f). We present only values of f for which alleles at targeting and target loci oscillate when recombination is PRDM9 (Red-queen dynamics). Each row within pairs corresponds to a different initial frequency $m^{0}$ of the specific allele: Either the non-PRDM9 allele is initially fixed and a rare PRDM9 mutant arises with frequency $m^{0}=0.01$ (*Top* row), or the PRDM9 allele is initially fixed and a rare non-PRDM9 mutant arises with frequency $m^{0}=0.99$ (*Bottom* row). Figures show which modifier allele goes to fixation given initial frequencies $p_{0}$ and $q_{0}$ of targeting and alleles $A_{1}$ and $B_{1}$. Red dots indicate that the non-PRDM9 allele goes to fixation whereas blue dots indicate that the PRDM9 allele goes to fixation. For each value of f, the probabilities of transition are summarized schematically under each pair of figures. Arrows represent transitions between types of recombination hotspots. A missing arrow indicates no transition. The three panels correspond to the ranges of values of f for which qualitatively different behavior is observed. Panel (*A*) is for f∈(0.10,0.29) which is characterized by PRDM9 never replacing non-PRDM9, and non-PRDM9 always replacing PRDM9. Panel (*B*) is for f∈(0.30,0.43) which is characterized by PRDM9 never replacing non-PRDM9 and non-PRDM9 replacing PRDM9 with a positive probability (47 to 71%). These two cases correspond to non-PRDM9 being the only evolutionarily stable strategy. Panel (*C*) is for f∈(0.44,0.56) which is characterized by PRDM9 replacing non-PRDM9 with probability 0.4 to 29% and non-PRDM9 replacing PRDM9 with probability between 49 to 58%. This last case corresponds to transitions between both types of recombination hotspots.

$m^{0}\approx 0$. Numerical analyses indicate that when the unspecific allele is resident and the specific allele is rare ($m^{0}\approx 0$), the specific allele almost never replaces the unspecific one (Fig. 3). Consequently, transitions from non-PRDM9 to PRDM9 recombination hotspots are not generally expected. An exception occurs near the upper end of the oscillatory region, when the reduction in fertility due to the absence of binding satisfies $0.44<f<f_{\mathrm{cyc}}\approx 0.57$ (Fig. 3*C*). In this parameter range, transitions to PRDM9 hotspots occur with probabilities between 0% and 29%, increasing with f.

#### PRDM9 resident and non-PRDM9 mutant:.

$m^{0}\approx 1$. Starting from the limit cycle, a rare unspecific allele replaces a resident specific allele when f is small, i.e., $0<f<\frac{1}{2}f_{\mathrm{cyc}}\approx 0.29$ (Fig. 3*A*). For intermediate values, $0.30\leq f<f_{\mathrm{cyc}}\approx 0.57$, replacement occurs only if one targeting allele is initially near fixation ($p^{0}\approx 0$ or $p^{0}\approx 1$), while $q^{0}$ can take arbitrary values (Fig. 3 *B* and *C*). Within the narrow interval 0.29<f≤0.32, the probability of replacement declines sharply from 1 to 0.43 (Fig. 3 *A* and *B*). For larger values of f, replacement probabilities increase again, reaching 0.58 at f=0.56 (Fig. 3*C*). Except when f is large (f>0.43), all observed transitions are toward non-PRDM9 hotspots, implying eventual replacement of PRDM9 hotspots.

#### Evolutionary stability of each type of hotspot.

Taken together, these results show that when f is small, $0<f<\frac{1}{2}f_{\mathrm{cyc}}\approx 0.29$, non-PRDM9 hotspots are the only evolutionarily stable outcome: Resident unspecific alleles are never replaced, whereas resident specific alleles are always replaced (Fig. 3*A*). For intermediate values, $\frac{1}{2}f_{\mathrm{cyc}}<f<f_{\mathrm{cyc}}$, non-PRDM9 hotspots remain uninvadable: Resident unspecific alleles are never replaced, whereas resident specific alleles are replaced in many cases, with probabilities of at least 43% per mutation at the targeting locus (Fig. 3*B*). Once established, non-PRDM9 hotspots cannot be replaced by PRDM9 ones, implying that eventual fixation of non-PRDM9 hotspots is only a matter of time.

An exception arises near the upper boundary of the oscillatory region, $0.44<f<f_{\mathrm{cyc}}\approx 0.57$ (Fig. 3*C*). In this regime, each hotspot type can sometimes replace the other: Non-PRDM9 hotspots are replaced by PRDM9 ones in 0 to 29% of cases, while PRDM9 hotspots are replaced in 49 to 58% of cases. Under these conditions, neither hotspot type is evolutionarily stable, favoring repeated transitions between PRDM9 and non-PRDM9 recombination hotspots.

### Increasing Difference in Crossover Between Symmetric and Asymmetric Binding:.

$\rho_{s}>\rho_{a}$.

#### Very small difference:.

**Fig. 4.**
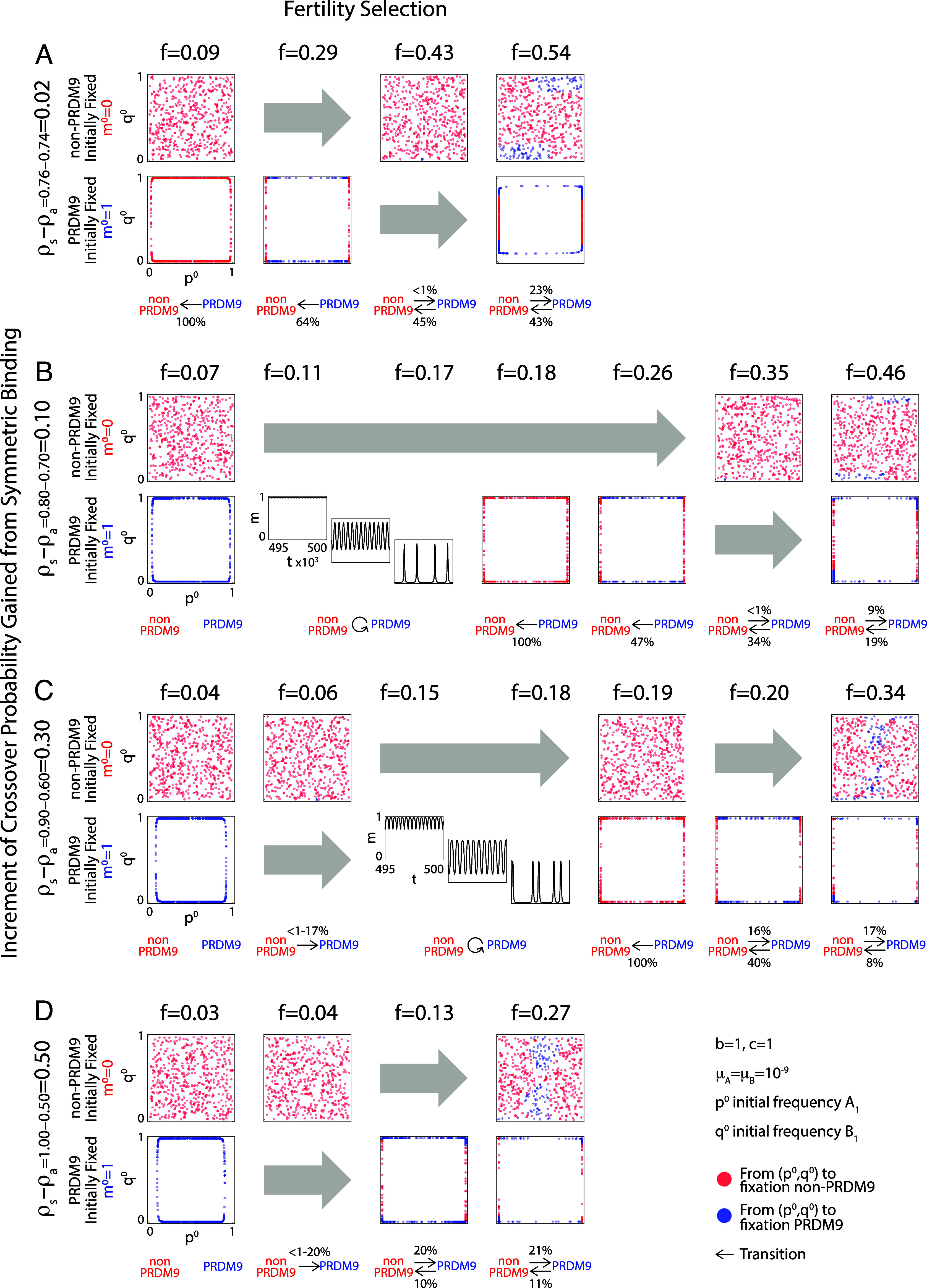
Transitions between types of recombination hotspots when symmetric binding is advantageous. The interpretation of each figure is the same as in Fig. 3. However, we identify a new qualitative behavior corresponding to the coexistence of both recombination hotspots due to the oscillation of allelic frequencies at the modifier locus. Because there is no replacement of one allele by the other we represent the change in frequency of allele PRDM9 over time (in thousands of generations) for the initial condition $\left(p^{0},q^{0}\right)=\left(0.9999,0.9999\right)$ in the last 5 thousand generations (from 495 to 500 thousand). Gray arrows indicate that the qualitative behavior remains the same in that range of f values. Each pair of rows corresponds to a given advantage of symmetric relative to asymmetric binding. Each panel corresponds to a different increments in crossover probability gained from symmetric binding: *A*. very small, *B*. small, *C*. moderate, and *D*. large differences.

$\rho_{s}-\rho_{a}=0.02$. This small difference does not alter the qualitative results obtained for $\rho_{s}=\rho_{a}$. When the reduction in fertility due to the absence of binding is small, 0.09≤f≤0.42, non-PRDM9 hotspots are the only evolutionarily stable hotspots and they consistently replace PRDM9 hotspots (Fig. 4*A*). When f is larger, 0.43≤f≤0.54, neither hotspot type is evolutionarily stable, and natural selection favors repeated transitions between the two types (Fig. 4*A*). Representative examples of the evolutionary dynamics identified here and below are shown in *SI Appendix*, Figs. S2–S6.

#### Small difference:.

$\rho_{s}-\rho_{a}=0.10$. The qualitative regimes observed in the previous section persist, but over shifted parameter ranges. In addition, two qualitatively new regimes emerge, namely i) no transitions between types of hotspot, and ii) both hotspot types are used with cyclical oscillations of their relative use. For small values of f (0.07≤f≤0.11), both hotspot types are evolutionarily stable and no transitions occur between them (Fig. 4*B*). At intermediate values (0.11≤f≤0.18), the two hotspot types can coexist through cyclical dynamics (Fig. 4*B*). Increasing the reduction in fertility f lowers the average use of PRDM9 hotspots and increases the amplitude of oscillations. For larger values (0.18≤f≤0.35), non-PRDM9 hotspots become the only evolutionarily stable type (Fig. 4*B*). Finally, when 0.35≤f≤0.46, neither hotspot type is evolutionarily stable and bidirectional transitions are favored by natural selection (Fig. 4*B*).

#### Moderate difference:.

$\rho_{s}-\rho_{a}=0.30$. The same qualitative regimes as observed in the previous section persist. In addition, a qualitatively new regime emerges, namely that PRDM9 hotspots are the only type of hotspot used. If 0.04≤f<0.06, both hotspot types are evolutionarily stable and no transitions occur (Fig. 4*C*). For 0.06≤f<0.15, PRDM9 hotspots are the only evolutionarily stable type, as PRDM9 can replace non-PRDM9 hotspots, albeit only from certain initial conditions (Fig. 4*C*). If 0.15≤f≤0.18, the two hotspot types coexist with oscillating frequencies. At f≈0.19, non-PRDM9 hotspots become the only evolutionarily stable type (Fig. 4*C*). If 0.20≤f≤0.34, neither hotspot type is evolutionarily stable and transitions between them are favored by natural selection (Fig. 4*C*).

#### Large difference:.

$\rho_{s}-\rho_{a}=0.50$. Such large differences eliminate some of the behaviors observed at lower values, notably i) non-PRDM9 hotspots are the only type of hotspots used and ii) both hotspot types are used with cyclical oscillations of their relative use. If f is very small (0.03≤f<0.04), both hotspot types are evolutionarily stable and no transitions occur (Fig. 4*D*). If 0.04≤f<0.13, PRDM9 hotspots are evolutionarily stable and can replace non-PRDM9 hotspots, although with low probability (Fig. 4*D*). If 0.13≤f≤0.27, neither hotspot type is evolutionarily stable, and transitions in both directions are favored (Fig. 4*D*).

To generate these results we assumed that mutation rates are low, $\mu_{A}=\mu_{B}=\mu =10^{-9}$, because then the range of parameter values where each behavior is observed is wide, facilitating its graphical presentation. Higher mutation rates do not change our findings qualitatively but the regions where each behavior is observed are compressed, i.e., transitions in behavior occur for smaller differences of $\rho_{s}-\rho_{a}$ (see *SI Appendix* for results with $\mu =10^{-7}$).

Intuition for these results can be gained from Eq. **4**. It shows that the per-generation change of the frequency m of the specific modifier allele $M_{1}$ is of the form $\overset{¯}{w}\Delta m\approx m\left(1-m\right)\frac{1}{4}bfT$, where the term T is independent of m. Therefore the modifier is under direct, though frequency-dependent, selection. If there is no crossover-resolution advantage of symmetric binding ($\rho_{s}=\rho_{a}$) Eq. **4** reduces to

[5] $$\begin{array}{c} \overset{¯}{w}\Delta m\approx m\left(1-m\right)\frac{1}{2}fb\rho_{a}\left[\frac{1}{2}\left(1-2p\right)\left(1-2q\right)+D_{\mathrm{AB}}\right]\;. \end{array}$$

The first term in the brackets shows that oscillations in allele frequencies at the targeting and target loci induce oscillations in modifier allele frequencies. The second term shows that linkage disequilibrium between alleles at the targeting and target loci—always negative when $f<f_{\mathrm{cyc}}$—leads to PRDM9 hotspots being selected against. Taken together, these two terms indicate that Δm is more often negative than positive.

This observation is consistent with our intuition. Because PRDM9 proteins preferentially bind one motif, they drive the overtransmission of the target motif that they bind less frequently. As a result, targeting alleles tend to become preferentially associated with mismatching targets, generating negative linkage disequilibrium between targeting and target sites ($D_{\mathrm{AB}}<0$ in the parameter region considered) (cf. 26). This preferential association between mismatching alleles yields slightly lower binding rates than would be achieved in the absence of any sequence recognition. Consequently, when there is no difference between symmetric and asymmetric binding, natural selection favors a mechanism that does not recognize any sequence—that is, non-PRDM9 recombination hotspots—because it results in higher overall recombination.

If there is a crossover-resolution advantage of symmetric binding ($\rho_{s}>\rho_{a}$), Eq. **4** can be used to derive an approximation for the selection coefficient of the specific modifier in the vicinity of hotspots (p,q≈1 or p,q≈0) and coldspots (p≈1,q≈0 or p≈0,q≈1). Because linkage disequilibrium between the targeting and target loci is negligible in these regions, the approximation is

[6a] $$\begin{array}{cc} & \frac{\overset{¯}{w}\Delta m}{m(1-m)}\\ & \approx \frac{1}{2}f\times \left\{\begin{array}{cc} {}[\frac{1}{2}b\rho_{a}+\frac{3}{4}b^{2}\left(\rho_{s}-\rho_{a}\right)] & \text{near hot},\\ {}[-\frac{1}{2}b\rho_{a}-\frac{1}{4}b^{2}\left(\rho_{s}-\rho_{a}\right)] & \text{near cold}, \end{array}\right. \end{array}$$

[6a] $$\begin{array}{cc} & =\frac{1}{2}f\times \left\{\begin{array}{cc} {}[\underset{\Delta B_{h}^{a}}{\underbrace{(\frac{1}{2}b-\frac{3}{4}b^{2})}}\rho_{a}+\underset{\Delta B_{h}^{s}}{\underbrace{\frac{3}{4}b^{2}}}\rho_{s}] & \text{near hot},\\ {}[\underset{\Delta B_{c}^{a}}{\underbrace{-(\frac{1}{2}b-\frac{1}{4}b^{2})}}\rho_{a}\underset{\Delta B_{c}^{s}}{\underbrace{-\frac{1}{4}b^{2}}}\rho_{s}] & \text{near cold}. \end{array}\right. \end{array}$$

We note that the difference in the probability of symmetric binding near hotspots between specific and unspecific modifiers is $b^{2}-\frac{1}{4}b^{2}=\frac{3}{4}b^{2}=\Delta B_{h}^{s}$. The difference in the probability of asymmetric binding near hotspots between specific and unspecific modifiers is $b-b^{2}-\left(\frac{1}{2}b+\frac{1}{4}b^{2}\right)=\frac{1}{2}b-\frac{3}{4}b^{2}=\Delta B_{h}^{a}$. Similarly, the difference in the probability of symmetric binding near coldspots between specific and unspecific modifiers is $0-\frac{1}{4}b^{2}=-\frac{1}{4}b^{2}=\Delta B_{c}^{s}$. The difference in the probability of asymmetric binding near coldspots between specific and unspecific modifiers is $0-\left(\frac{1}{2}b-\frac{1}{4}b^{2}\right)=\Delta B_{c}^{a}$.

Eq. **6a** shows that when symmetric binding increases the probability of crossovers, selection in favor of specific modifiers near hotspots is stronger than selection against them near coldspots. Eq. **6b** further shows that the net positive selection for specific modifiers arises because specific modifiers gain more symmetric bindings near hotspots than they lose near coldspots ($\Delta B_{h}^{s}>-\Delta B_{c}^{s}$); the net gain is $\Delta B_{h}^{s}+\Delta B_{c}^{s}=\frac{1}{2}b^{2}$. Because the dynamics of targeting and target loci are oscillatory, with hotspots and coldspots alternating over time, specific modifiers experience periodic phases of positive and negative selection. Our results indicate that when symmetric binding confers a crossover-resolution advantage over asymmetric, selection in favor of the specific modifier is stronger than selection against it. This occurs because, in hotspots, the specific modifier produces more symmetric bindings than the unspecific modifier, while in coldspots the unspecific modifier produces more symmetric bindings than the specific one, but not enough to offset the excess produced by the specific modifier in hotspots. As a result, when averaged over a full cycle, specific modifiers generate more symmetric bindings than unspecific modifiers.

Our model does not assume that specific targeting increases symmetric binding. Instead, it demonstrates that specific targeting increases the net production of symmetric bindings relative to unspecific targeting over time when the targeting and target loci oscillate, as they do for PRDM9 loci. Importantly, this outcome emerges from the dynamics of the model rather than from any explicit assumption.

In summary, the intuition derived from the expressions above shows that PRDM9 has an intrinsic fitness disadvantage due to the preferential association of mismatching targeting and target alleles (negative linkage disequilibrium), which reduces the probability of binding relative to its non-PRDM9 alternative. However, when symmetric binding has a crossover-resolution advantage, PRDM9 gains a fitness advantage because it produces more symmetric bindings in hotspots than it loses in coldspots, relative to a non-PRDM9 competitor. The selective advantage resulting from symmetric binding can therefore outweigh the intrinsic fitness disadvantage caused by mismatched associations. Furthermore, for certain values of the relative advantage of symmetric over asymmetric binding, the disadvantage of PRDM9 hotspots due to lower binding probability can balance the advantage derived from higher net production of symmetric binding, allowing both hotspot mechanisms to coexist with the frequency in which each type is used oscillating.

## Discussion

In nature, there are two types of recombination hotspots: PRDM9 hotspots and non-PRDM9 recombination hotspots (12, 23). PRDM9 hotspots are determined by proteins that bind specific DNA sequences at which recombination is initiated (12) (Fig. 1). Non-PRDM9 recombination hotspots, by contrast, are not determined by proteins that bind specific sequences; instead, recombination is initiated in open chromatin regions independently of the underlying DNA sequence (23) (Fig. 1). Although they rely on different mechanisms, both types of hotspots achieve the same functional goal of initiating recombination and facilitating proper chromosomal segregation during meiosis. Some species use PRDM9 hotspots, others use non-PRDM9 hotspots (13), and still others use both types simultaneously (40, 41). Knockout experiments in species that normally use PRDM9 hotspots show that recombination can still be carried out by non-PRDM9 hotspots (16, 36–38). If the two types of hotspots represent alternative ways of achieving recombination, why does evolution favor the origin and maintenance of one type in the presence of the other? What evolutionary forces favor PRDM9 hotspots in some species but not in others? The evolution of recombination hotspots therefore remains an open evolutionary puzzle.

To address this question, we formulate and analyze a population genetic model of a modifier locus that changes the specificity of the coevolutionary interaction between a targeting locus and its target sites. The production of specific proteins results in crossover initiation following recognition of a DNA-sequence motif. These specific proteins correspond to the action of PRDM9 hotspots, which exhibit erosion of hotspots through overtransmission of the allele that does not break. By contrast, the production of unspecific proteins results in crossover initiation independently of any DNA-sequence motif. Unspecific proteins correspond to the action of non-PRDM9 hotspots, which do not exhibit hotspot erosion, because both alleles are equally likely to break and are therefore transmitted equally. These unspecific proteins can also be interpreted as the absence of production of specific proteins. In our model, the coevolutionary interaction between the targeting and target loci is driven by two factors: i) fertility loss due to the absence of crossovers, and ii) differences between symmetric and asymmetric bindings in their likelihood of resolving as crossovers. Although we discuss the model in terms of crossovers, it applies equally to any effects that binding, double-strand breaks, chromosome pairing, and crossovers may have on individual fertility.

Our results show that when symmetric and asymmetric bindings are equally likely to resolve as crossovers ($\rho_{s}=\rho_{a}$), or only slightly more likely to do so ($\rho_{s}-\rho_{a}$ small), non-PRDM9 hotspots are the only evolutionarily stable type. This holds across a wide range of values of fertility loss due to lack of crossover, f (Figs. 3 and 4*A*). For high values of f, however, neither PRDM9 hotspots nor non-PRDM9 hotspots are evolutionarily stable, and recurrent transitions between the two types occur (Figs. 3 and 4*A*). The fact that only non-PRDM9 hotspots are evolutionarily stable in these cases has two implications: i) PRDM9 hotspots cannot evolve in the presence of an alternative, redundant non-PRDM9 recombination mechanism, and ii) natural selection favors transitions from PRDM9 hotspots to non-PRDM9 hotspots, but not in the opposite direction. This unidirectionality implies that, over evolutionary time, PRDM9 hotspots should be lost in all clades in which the fitness gain of symmetric binding is small or absent.

**Fig. 5.**
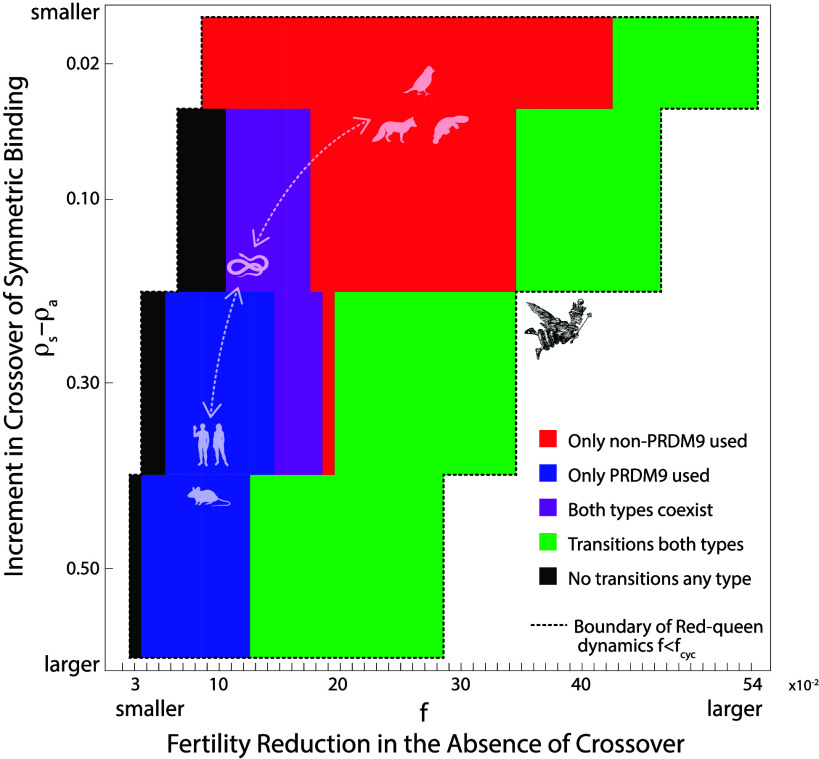
Types of hotspots used in different regions of the parameter space. The horizontal axis shows the fertility reduction in the absence of crossover f. The vertical axis shows the crossover-resolution advantage of symmetric binding$\rho_{s}-\rho_{a}$. Each color represents a different qualitative behavior: i) non-PRDM9 is the only evolutionarily stable strategy and it is the only type of hotspot predicted to evolve in the red region; ii) PRDM9 is the only evolutionarily stable strategy and it is the only type of hotspot predicted to evolve in the blue region; iii) both types of hotspots coexist with the frequency of each hotspot oscillating over evolutionary time in the purple regions; iv) both types of hotspots can replace each other and a rapid turn-over between types of hotspots is likely to evolve in the green region; v) none of the two types of hotspots can replace the other and no transitions between types of hotspots should be observed in the black region. Silhouettes represent species with the type of recombination hotspot predicted by that region of the parameter space. Dotted lines demarcate the region where Red-queen dynamics between targeting and target site are expected. Parameters are b=c=1 and $\mu =10^{-9}$.

When symmetric bindings are more likely to resolve as crossovers than asymmetric ones ($\rho_{s}-\rho_{a}$ large), both PRDM9 hotspots and non-PRDM9 hotspots can coexist within a single population, with regular oscillations in the usage of each type (Fig. 4 *B* and *C*). The greater the crossover-resolution advantage of symmetric binding ($\rho_{s}-\rho_{a}$), the higher the average usage of PRDM9 hotspots and the lower the amplitude of the oscillations, which vanishes for very large $\rho_{s}-\rho_{a}$ (Fig. 4*D*). Conversely, the greater the reduction in fertility due to lack of crossovers f, the lower the average usage of PRDM9 hotspots and the higher the amplitude of the oscillations (Fig. 4 *B* and *C*). When the crossover advantage of symmetric binding is large, PRDM9 hotspots can be the only evolutionarily stable type of hotspot. This occurs for low values of f (Figs. 4 *C* and *D* and 5). For very low values of f, no transitions between PRDM9 hotspots and non-PRDM9 hotspots occur, and both types are evolutionarily stable. For moderate or high values of f, recurrent transitions between the two hotspot types are observed (Figs. 4 and 5).

The intuitive reason why PRDM9 hotspots are sometimes favored by natural selection lies in the sequence-specific targeting mechanism employed by PRDM9. On the one hand, the specificity of the PRDM9 protein leads to overtransmission of target motifs that break less frequently. This promotes preferential associations between PRDM9 targeting alleles and target sequences that are not recognized (i.e. negative linkage disequilibrium), which in turn results in slightly lower crossover rates than would occur in the absence of sequence recognition. Because crossovers promote fertility by ensuring proper chromosomal segregation, PRDM9 hotspots have an intrinsic evolutionary disadvantage compared to non-PRDM9 recombination mechanisms. On the other hand, PRDM9 specificity leads to higher production of symmetric binding events in hotspot genetic backgrounds and lower production of symmetric binding events in coldspot backgrounds when compared to a non-PRDM9 alternative. Importantly, the excess of symmetric binding produced in hotspots exceeds its deficiency in coldspots, thus resulting in a net excess of symmetric binding over time. When symmetric bindings are more likely to be resolved as crossovers than asymmetric bindings, this net excess allows PRDM9 hotspots to balance—or even overcome—their evolutionary disadvantage arising from preferential association with mismatching targets.

To summarize, our key results and their intuitive explanations are as follows: 1. When symmetric and asymmetric bindings are equally likely to resolve as crossovers, natural selection favors non-PRDM9 hotspots in most cases. This occurs because the preferential association between mismatching targeting and target alleles is inherent to PRDM9 hotspots. 2. When symmetric bindings are more likely to resolve as crossovers than asymmetric, natural selection favors either: a) coexistence of PRDM9 and non-PRDM9 hotspots, when the fitness benefit of producing more symmetric bindings balances the fitness cost of associating with mismatching alleles; or b) PRDM9 hotspots, when the fitness benefit of producing more symmetric bindings outweighs the fitness cost of associating with mismatching alleles. Both results are unexpected because, given that our model assumes equal genotype-averaged binding probabilities for both types of hotspots, we expected natural selection to be indifferent between them. Notably, both the preferential associations between mismatching alleles and the overproduction of symmetric bindings are not explicit assumptions of our model, but instead emerge from the analysis of the underlying dynamical system.

These findings are relevant because they can explain the gains, losses, and coexistence of both types of hotspots, behaviors that have been documented by empirical work but so far remained unexplained. The absence of PRDM9 hotspots in plants and fungi, together with their presence in vertebrates, suggests a transition from non-PRDM9 hotspots to PRDM9 hotspots in the common ancestor of vertebrates (39, 40) (Fig. 2). This interpretation is supported by the fact that knockouts of the gene PRDM9 in mice and rats do not eliminate recombination, but instead result in a reversion to a seemingly redundant ancestral non-PRDM9 recombination hotspot mechanism—an effect that is also observed in humans who are homozygous for nonfunctional PRDM9 alleles (16, 36–38). According to our model, the transition from non-PRDM9 hotspots to PRDM9 hotspots in the common ancestor of vertebrates can be explained if the crossover-resolution advantage of symmetric binding was sufficiently large relative to the fitness loss caused by a lack of crossovers in that ancestor (Fig. 5).

Although PRDM9 hotspots are pervasive throughout the vertebrate phylogeny, multiple independent losses have been documented—for example in the common ancestor of crocodilians and birds, as well as in canids (13, 35) (Fig. 2). The long-term maintenance of PRDM9 hotspots observed in many vertebrate lineages is consistent with our model when the crossover-resolution advantage of symmetric binding is large relative to the fitness loss caused by a lack of crossovers (Fig. 5). Conversely, the repeated losses of PRDM9 hotspots are consistent with our model when the crossover-resolution advantage of symmetric binding is small relative to the fitness loss associated with a lack of crossovers (Fig. 5). Furthermore, recent empirical evidence suggests the coexistence of both types of hotspots in many vertebrate species (40, 41). This observation is consistent with our model when crossover-resolution advantage of symmetric binding balances fitness losses due to a lack of crossovers (Fig. 5). Our model predicts the coexistence of PRDM9 hotspots and non-PRDM9 hotspots with their relative use fluctuating over evolutionary time.

Finally, our model generates testable predictions about the selective regimes that favor the evolution of each type of hotspot. At present, there are no comparative data that quantify the fitness impact of lack of crossovers or differences in crossover resolution between types of bindings. However, our model provides testable predictions that can guide future data collection across species. Specifically, it predicts that in species using primarily PRDM9 hotspots (e.g. humans and mice), the fitness disadvantage associated with lack of crossovers should be low relative to the crossover-resolution advantage of symmetric binding (Fig. 5). In contrast, in species not using PRDM9 hotspots (e.g. birds and canids), the fitness disadvantage of lack of crossovers should be high relative to the crossover-resolution advantage of symmetric binding (Fig. 5). In species using both types of hotspots (e.g. snakes), the fitness disadvantage of a lack of binding and the crossover-resolution advantage of symmetric binding should take intermediate values (Fig. 5). Empirical tests of these predictions will help clarify the role of PRDM9 in the evolution of recombination.

Our model relies on a small set of key parameters, namely the reduction f in fertility of individuals that do not experience at least one crossover, and the probabilities $\rho_{s}$ and $\rho_{a}$ that symmetric and asymmetric bindings, respectively, are resolved as crossovers. The choice of these parameters closely follows the empirical literature. Proper segregation typically requires at least one crossover per chromosome arm, a constraint often referred to as crossover assurance (55–58), and failure to form a crossover greatly increases the risk of nondisjunction and aneuploidy, often resulting in embryonic lethality or the production of nonviable gametes (55–58). Empirical studies across taxa show that individuals lacking crossovers frequently exhibit meiotic errors and reduced reproductive success (47, 48). Consistent with these observations, mutations in recombination-related genes—including PRDM9—have been linked to infertility phenotypes such as meiotic arrest and azoospermia in mammals (59, 60). Together, these findings support the view that the absence of crossovers imposes a fitness cost, making reliable crossover formation essential for fertility.

Studies of hybrid sterility in mice indicate that symmetric PRDM9 binding is not strictly required for crossover formation (21, 45, 52), but it increases the likelihood that double-strand breaks are resolved as crossovers by promoting efficient homolog engagement and timely repair (22, 44, 45). Because crossovers are essential for proper chromosome segregation, differences in the probability of crossover resolution translate directly into differences in fitness (22, 45). Although the precise mechanisms by which symmetric binding enhances crossover formation remain unclear, several nonmutually exclusive explanations have been proposed: i) symmetric binding may promote chromatin decondensation on the homolog used as a repair template, ii) it may help recruit or correctly position the homolog at the chromosome axis, thereby reducing the homology search space, and iii) PRDM9 molecules bound to each homolog may interact directly to facilitate homolog engagement (44, 45, 52, 61, 62). Whereas the qualitative impact of these parameters on fertility is well established, there is little quantitative information about their magnitude, and no comparative data across species. For that reason our results explore the entire space of these parameter values.

We provide an example of why the crossover-resolution advantage of symmetric binding may vary across taxa. One of the ideas to explain why symmetric binding may enhance crossover formation is that it reduces the homology search space (44, 45, 52, 61, 62). If this is the case, the impact of symmetric binding is expected to depend on chromosomal architecture, particularly average chromosome size. Species with smaller average chromosomes, such as birds (e.g. herons have an average chromosome size of 38 mega base-pairs, 63) and canids (e.g. wolves ∼70 Mb, 63), already operate within a relatively constrained search space and thus derive limited benefit from further reductions. In contrast, species with larger chromosomes, including noncanid mammals (e.g. humans ∼149Mb, 63), experience a substantially larger search space and therefore benefit most from its reduction. Species with intermediate chromosome sizes, such as snakes (e.g. rattlesnakes ∼95 Mb, 63), experience a moderately larger search space and are expected to derive an intermediate benefit. In this framework, large average chromosome sizes are expected to favor the evolution of PRDM9 hotspots, whereas small chromosomes are more consistent with non-PRDM9 hotspots.

Our model assumes an infinite population and therefore ignores random genetic drift. Here, we discuss why we nevertheless expect it to yield important insights into the evolution of PRDM9 recombination hotspots under more realistic, finite-population conditions. In our model we often find oscillations of allelic frequencies. Because these oscillations often bring one of the alleles close to fixation, elimination of rare alleles by random drift will inevitably occur even in large finite populations. In finite populations, random genetic drift will therefore tend to truncate the regular oscillatory dynamics at the targeting and target loci by accelerating fixation or loss during hotspot–coldspot transitions. This renders the dynamics more irregular and shifts the precise parameter boundaries between evolutionary regimes (see ref. 26 for targeting–target dynamics in finite populations). However, these effects primarily influence the timing and smoothness of transitions rather than the underlying qualitative behavior. In particular, although oscillations at a given target may be reduced or prematurely terminated, the presence of multiple potential targets allows hotspots to re-emerge at new genomic locations, leading to continued hotspot turnover over evolutionary time (26).

A key finding of our work is that, in contrast to prominent classical modifier models, selection on the modifier of specificity is direct and strong (*SI Appendix*). Moreover, the strength of selection on the modifier is maximized when polymorphism at the primary loci is eliminated by drift: Selection in favor of PRDM9 is strongest near hotspots, whereas selection in favor of non-PRDM9 is strongest near coldspots. Because selection is strong in our model, modifier evolution in finite populations is expected to proceed largely as predicted by the deterministic model, albeit with greater stochasticity. Taken together, these considerations suggest that while finite population size introduces noise and irregularity, it does not overturn the central predictions of the model regarding modifier evolution, thus supporting the relevance of our deterministic analysis for understanding the evolution of PRDM9 in natural populations.

There are additional selective forces acting on PRDM9-guided and non-PRDM9 recombination hotspots that merit consideration. For example, fitness differences associated with the genomic regions in which each type of recombination hotspot is located, given that non-PRDM9 hotspots are enriched in promoter regions, or differences in the recombination landscape produced by each mechanism, as non-PRDM9 hotspots are associated with a stable landscape lacking spatial or temporal variation. Investigation of the effects of such additional selective forces therefore constitutes an interesting direction for future research. If such forces operate, they are expected to act in conjunction with, rather than independently of, the intrinsic evolutionary forces captured by our model. Our framework thus provides a principled baseline against which additional selective hypotheses can be formally evaluated.

## Supplementary Material

Appendix 01 (PDF)

## Data Availability

The *Mathematica* (Wolfram) notebook referred to above and in *SI Appendix* has been deposited in GitHub (https://github.com/fubeda72/OriginPRDM9) (53).
