# Supplementary material for: On the origin of PRDM9-guided recombination hotspots
Source: Proc Natl Acad Sci U S A. 2026 Jun 25;123(26):e2535682123. doi: 10.1073/pnas.2535682123 (PMC13320716; doi:10.1073/pnas.2535682123)
Supplement: Supplementary file 1 — Appendix 01 (PDF) [file pnas.2535682123.sapp.pdf]

Supporting Information for

On the origin of PRDM9-guided recombination hotspots

Francisco Úbeda, Reinhard Bürger and Frederic Fyon

Francisco Úbeda;
E-mail: f.ubeda@rhul.ac.uk

This PDF file includes:

Supporting text
Figs. S1 to S9
Table S1
SI References

Supporting Information Text

1. Appendix

In this appendix we provide a detailed derivation and treatment of the model employed in the main text. We also include some additional results and graphical illustrations. The supplementary *Mathematica* notebook contains the code and the derivations to verify the results presented here.

A. General model. We model the interaction between three loci: the modifier locus M, the targeting locus A and the target locus B. Locus A may carry alleles A_k (where $k \in \{1, 2\}$), each encoding a protein that attempts to bind a DNA base-pair motif at locus B. Locus B may carry alleles B_m (where $m \in \{1, 2\}$), each corresponding to a sequence motif for which the binding protein may show differential specificity. Locus M carries alleles M_i (where $i \in \{0, 1\}$), each contributing to a genotype that determines whether the binding protein binds both motifs with equal probability, thus showing no specificity, or binds one motif with higher probability than the other, thus showing specificity.

A.1. Binding probabilities. Let $x_{i,k,m}$ be the frequency of haplotype $M_iA_kB_m$ in gametes, where $0 \le x_{i,k,m} \le 1$ and $\sum_{i,k,m} x_{i,k,m} = 1$. Random union of gametes results in a zygote with genotype $\frac{M_iA_kB_m}{M_jA_lB_n}$ with frequency $x_{i,k,m}x_{j,l,n}$. A graphical representation of the basic model is displayed in Fig. S1.

Individuals homozygous for the modifier allele M_1 produce proteins with specificity, that is proteins produced by locus A behave like PRDM9 proteins. The specific protein A_k binds matching (B_k) and non-matching (B_m) target motifs with probabilities $b_{k,m}$, such that $0 \le b_{k,m} < b_{k,k} \le 1$, where $m \ne k$. For simplicity, henceforth we assume that $b_{k,m} = 0$ and $b_{k,k} = b$, where $0 < b \le 1$. The probability that one of the specific proteins binds target B_m is $\bar{b}_{kl,m} = \frac{1}{2}b_{k,m} + \frac{1}{2}b_{l,m}$. Targeting proteins attempt to bind both targets. We assume:

(i) Two bindings, known as symmetric binding, occur with probability

$$b^s_{kl,mn} = \bar{b}_{kl,m}\bar{b}_{kl,n} \, , \tag{1}$$

i.e., the two binding events occur independently.

(ii) Asymmetric binding, i.e., binding at one target allele but not the other, occurs with probability

$$b^a_{kl,mn} = \frac{1}{2}\bar{b}_{kl,m}\left(1 - \bar{b}_{kl,n}\right) + \frac{1}{2}\left(1 - \bar{b}_{kl,m}\right)\bar{b}_{kl,n} \, . \tag{2}$$

Individuals homozygous for the modifier allele M_0 produce proteins with no specificity. The unspecific protein A_k binds matching (B_k) and non-matching (B_m) target motifs with equal probability $\beta_{k,m} = \beta_{k,k}$. For simplicity, henceforth we assume $\beta_{k,m} = \beta_{k,k} = \beta$, where $0 < \beta < 1$. The probability that one of the unspecific proteins binds target B_m is $\bar{\beta}_{kl,m} = \frac{1}{2}\beta_{k,m} + \frac{1}{2}\beta_{l,m} = \beta$. Targeting proteins attempt to bind both targets. We assume:

(i) Symmetric binding occurs with probability

$$\beta^s_{kl,mn} = \bar{\beta}_{kl,m}\bar{\beta}_{kl,n} = \beta^2 \, , \tag{3}$$

i.e., the two binding events occur independently.

(ii) Asymmetric binding occurs with probability

$$\beta^a_{kl,mn} = \frac{1}{2}\bar{\beta}_{kl,m}\left(1 - \bar{\beta}_{kl,n}\right) + \frac{1}{2}\left(1 - \bar{\beta}_{kl,m}\right)\bar{\beta}_{kl,n} = \beta(1-\beta) \, . \tag{4}$$

Individuals with genotype M_iM_j produce specific proteins with probability α_{ij}, where we assume $\alpha_{00} = 0$, $\alpha_{11} = 1$, $\alpha_{10} = \alpha_{01} = \frac{1}{2}$, i.e., absence of dominance. Then the probability that one of the proteins encoded by the targeting locus A binds target B_m is

$$\bar{B}_{ij,kl,m} = (1 - \alpha_{ij})\bar{\beta}_{kl,m} + \alpha_{ij}\bar{b}_{kl,m} \, . \tag{5}$$

Thus, $\bar{B}_{00,kl,m} = \bar{\beta}_{kl,m} = \beta$ and $\bar{B}_{11,kl,m} = \bar{b}_{kl,m}$.

Analogously, we define the probabilities of symmetric and asymmetric binding by

$$B^s_{ij,kl,mn} = (1 - \alpha_{ij})\beta^s_{kl,mn} + \alpha_{ij}b^s_{kl,mn} \, , \tag{6a}$$

$$B^a_{ij,kl,mn} = (1 - \alpha_{ij})\beta^a_{kl,mn} + \alpha_{ij}b^a_{kl,mn} \, , \tag{6b}$$

respectively. Straightforward calculations show that the symmetric and asymmetric binding probabilities satisfy the following relations:

$$B^s_{ii,kl,mn} = \bar{B}_{ii,kl,m}\bar{B}_{ii,kl,n} \, , \tag{7a}$$

$$B^s_{01,kl,mn} = \tfrac{1}{2}B^s_{00,kl,mn} + \tfrac{1}{2}B^s_{11,kl,mn} \, , \tag{7b}$$

$$B^a_{ii,kl,mn} = \tfrac{1}{2}\bar{B}_{ii,kl,m}(1 - \bar{B}_{ii,kl,n}) + \tfrac{1}{2}\bar{B}_{ii,kl,n}(1 - \bar{B}_{ii,kl,m}) \, , \tag{7c}$$

$$B^a_{01,kl,mn} = \tfrac{1}{2}B^a_{00,kl,mn} + \tfrac{1}{2}B^a_{11,kl,mn} \, . \tag{7d}$$

Table S1. Symmetric and asymmetric binding probabilities of the homozygous modifier genotypes. The asymmetric binding probabilities are given in the form 'total binding probability - symmetric binding probability'. Note that symmetric binding occurs with positive probability ($\frac{1}{4}b^2$) even if targeting and target locus are heterozygous. The corresponding binding probabilities of the heterozygous genotype M_0M_1 are the arithmetic means of the homozygous values; see Eq. (7). For $\beta = \frac{1}{2}b$ the binding probabilities in Fig. 4 of the main text are obtained.

Symmetric binding probabilities of M_1M_1:

$$B^s_{11,11,11} = B^s_{11,22,22} = b^2$$
$$B^s_{11,11,22} = B^s_{11,22,11} = 0$$
$$B^s_{11,11,12} = B^s_{11,11,21} = B^s_{11,22,12} = B^s_{11,22,21} = 0$$
$$B^s_{11,12,11} = B^s_{11,21,11} = B^s_{11,12,22} = B^s_{11,21,22} = \tfrac{1}{4}b^2$$
$$B^s_{11,12,12} = B^s_{11,21,12} = B^s_{11,12,21} = B^s_{11,21,21} = \tfrac{1}{4}b^2$$

Symmetric binding probabilities of M_0M_0:

$$B^s_{00,kl,mn} = \beta^2 \quad \text{for all } k,l,m,n \in \{1,2\}$$

Asymmetric binding probabilities of M_1M_1:

$$B^a_{11,11,11} = B^a_{11,22,22} = b - b^2$$
$$B^a_{11,11,22} = B^a_{11,22,11} = 0$$
$$B^a_{11,11,12} = B^a_{11,11,21} = B^a_{11,22,12} = B^a_{11,22,21} = \tfrac{1}{2}b$$
$$B^a_{11,12,11} = B^a_{11,21,11} = B^a_{11,12,22} = B^a_{11,21,22} = \tfrac{1}{2}b - \tfrac{1}{4}b^2$$
$$B^a_{11,12,12} = B^a_{11,21,12} = B^a_{11,12,21} = B^a_{11,21,21} = \tfrac{1}{2}b - \tfrac{1}{4}b^2$$

Asymmetric binding probabilities of M_0M_0:

$$B^a_{00,kl,mn} = \beta - \beta^2 \quad \text{for all } k,l,m,n \in \{1,2\}$$

In particular, the binding probabilities of the heterozygous genotype M_0M_1 are the arithmetic means of those of the homozygous genotypes. The relation in Eq. (7a) is analogous to the definitions in Eq. (1) and Eq. (3), and Eq. (7c) is analogous to the definitions in Eq. (2) and Eq. (4). These binding probabilities are specified in Table S1 and, if $\beta = \frac{1}{2}b$, in Fig. 4 of the main text.

For the derivation and the formulation of the recursion equations, we will need

$$B^{a*}_{ij,kl,mn} = \bar{B}_{ij,kl,m} - B^s_{ij,kl,mn}\,. \qquad [8]$$

This is the probability that the genotype M_iM_j causes (asymmetric) binding of A_k or A_l at the target allele B_m but no binding at B_n (even if there is a match). If $j = i$, then $B^{a*}_{ii,kl,mn} = \bar{B}_{ii,kl,m}(1 - \bar{B}_{ii,kl,n})$. Straightforward calculations confirm the expected result

$$B^a_{ij,kl,mn} = \tfrac{1}{2}B^{a*}_{ij,kl,mn} + \tfrac{1}{2}B^{a*}_{ij,kl,nm}\,. \qquad [9]$$

Finally, the probability of no binding is

$$B^0_{ij,kl,mn} = 1 - B^a_{ij,kl,mn} - B^s_{ij,kl,mn} = 1 - \frac{1}{2}\left(\bar{B}_{ij,kl,m} + \bar{B}_{ij,kl,n}\right), \qquad [10]$$

where $\frac{1}{2}\left(\bar{B}_{ij,kl,m} + \bar{B}_{ij,kl,n}\right)$ is the (total) probability of binding.

In the supplementary *Mathematica* notebook it is shown that the total binding probability of M_0M_0, averaged over all possible frequencies of all possible two-locus genotypes A_kB_m, equals the corresponding binding probability of M_1M_1 if and only if $\beta = \frac{1}{2}b$. For the specific modifier genotype M_1M_1, this *genotype-averaged total binding probability* is obtained by integrating $\sum_{k,l,m,n}(B^a_{11,kl,mn} + B^s_{11,kl,mn})x_{1,k,m}x_{1,l,n} = b\big(x_{1,1,1} + (x_{1,1,2} + x_{1,2,2})(x_{1,2,1} + x_{1,2,2})\big)$ with respect to the four targeting/target locus frequencies subject to the constraints $x_{1,k,m} \geq 0$ and $\sum_{k,m} x_{1,k,m} = 1$. After multiplication by 6 (because the volume of the gametic frequency space is $\frac{1}{6}$) we obtain a genotype-averaged total binding probability of $\frac{1}{2}b$. For the unspecific modifier, the respective probability is β because its binding probability is β for all genotypes (Table S1). Therefore, the choice $\beta = \frac{1}{2}b$, adopted throughout this paper, yields equal genotype-averaged total binding probabilities for the specific (M_1M_1) and the unspecific (M_0M_0) phenotypes. Potential costs or benefits of a modifier causing unspecific binding could be included by changing β.

We further remark that for the specific modifier genotype M_1M_1, the total binding probabilities are the same as the binding probabilities in the models studied by (1) and (2), who ignored symmetric binding and assumed that, given a match, binding occurs at only one of the two target motifs.

A.2. Double strand breaks, conversion and selection. We assume that both targeting alleles show the same level of expression producing a pool of targeting proteins with equal representation of those encoded by each allele. A double-strand break (DSB) initiates recombination, and the chromatid that breaks is repaired using its homologous chromatid as a template (Fig. S1). Let γ_a be the probability of a DSB and a correct pairing given an asymmetric binding, and let γ_s be the corresponding probability

given a symmetric binding. During the repair process there may be a crossover event in or near the target locus. We denote by r_a and r_s the probability of a crossover conditional on a DSB initiated by asymmetric and by symmetric pairing, respectively. We assume $0 \leq r_a \leq r_s \leq 1$ and $r_s > 0$.

During the repair process the allelic motif that breaks is converted into the allelic motif that does not break with probability c, where $0 \leq c \leq 1$. We note that biased conversion and segregation costs are independent of any recombination event between modifier, targeting, or target locus. Consistent with empirical observations and previous work (for references, see the main text), we assume that individuals not undergoing a DSB and crossover at the target locus may have defective chromosomal segregation, producing non-viable gametes with probability f, where $0 < f \leq 1$ (Fig. S1). The fitness of individuals experiencing crossover is 1, and the fitness of individuals not experiencing a DSB and crossover is $1 - f$. Therefore, the expected fitnesses of individuals experiencing no binding, asymmetric binding, and symmetric binding are,

$$F^0 = 1 - f\,, \quad [11a]$$

$$F^a = \gamma_a(r_a + (1 - r_a)(1 - f)) + (1 - \gamma_a)(1 - f) = 1 - f(1 - \gamma_a r_a)\,, \quad [11b]$$

$$F^s = \gamma_s(r_s + (1 - r_s)(1 - f)) + (1 - \gamma_s)(1 - f) = 1 - f(1 - \gamma_s r_s)\,, \quad [11c]$$

respectively. These fitnesses depend only on the compound parameters

$$\rho_a = \gamma_a r_a \text{ and } \rho_s = \gamma_s r_s\,, \quad [12]$$

so that we obtain $F^a = 1 - f(1 - \rho_a)$ and $F^s = 1 - f(1 - \rho_s)$. It follows that $F^a - F^0 = f\rho_a \geq 0$ and $F^s - F^a = f(\rho_s - \rho_a) \geq 0$.

It is important to note that instead of the parameters f, ρ_a, and ρ_s, we can use the fitnesses F^0, F^a, and F^s as independent parameters. Thus, for our model it is sufficient to have the fitnesses of genotypes experiencing no DSB (no binding), a DSB initiated by asymmetric binding, and a DSB initiated by symmetric binding.

It follows that the mean fitness of the population is

$$\bar{w} = \sum_{i,k,m} \sum_{j,l,n} \Big(F^0 B^0_{ij,kl,mn} + F^a B^a_{ij,kl,mn} + F^s B^s_{ij,kl,mn} \Big) x_{i,k,m} x_{j,l,n}\,. \quad [13]$$

Finally, we note that if $\rho_s = \rho_a$, then $F^s = F^a$. Therefore, if the specific modifier allele M_1 is fixed, the model reduces formally to that studied in Úbeda et al (1, 2), which ignored symmetric binding but assumed the same total binding probabilities.

B. The recursion equations for the gamete frequencies. We start by considering the effects of conversion. In case of symmetric binding, and independently of the genotype $M_i M_j$, the gamete $A_k B_m$ is maintained after conversion in a genotype $\frac{A_k B_m}{A_l B_n}$ with probability $\frac{3}{4}$, and it is generated by conversion from a genotype $\frac{A_k B_n}{A_l B_m}$ with probability $\frac{1}{4}$. Therefore, under symmetric binding the frequency of gametes $M_i A_k B_m$ after conversion (produced by all possible zygotes $\frac{M_i A_k B_m}{M_j A_l B_n}$) is

$$cF^s \sum_{j,l,n} \frac{3}{4} B^s_{ij,kl,mn} x_{i,k,m} x_{j,l,n} + cF^a \sum_{j,l,n} \frac{1}{4} B^s_{ij,kl,nm} x_{i,k,n} x_{j,l,m}\,. \quad [14]$$

In case of asymmetric binding, the frequency of $M_i A_k B_m$ after conversion is

$$cF^a \sum_{j,l,n} \frac{1}{4} B^{a*}_{ij,kl,mn} x_{i,k,m} x_{j,l,n} + cF^a \sum_{j,l,n} \frac{1}{2} B^{a*}_{ij,kl,nm} x_{i,k,m} x_{j,l,n} + cF^a \sum_{j,l,n} \frac{1}{4} B^{a*}_{ij,kl,nm} x_{i,k,n} x_{j,l,m}\,. \quad [15]$$

The first two terms in Eq. (15) arise by using Eq. (9) and noting that in case of a break at B_m the proportion of gametes $M_i A_k B_m$ is reduced by $\frac{1}{2}$, whereas in case of a break at B_n this proportion remains unchanged. The third term in Eq. (15) counts gametes $M_i A_k B_m$ produced by conversion in zygotes of type $M_i A_k B_n / M_j A_l B_m$.

By combining the case of no conversion (which has probability 1 if there is no binding, and $1 - c$ otherwise) with that of conversion, i.e., Eq. (14) and Eq. (15), we obtain the frequency $x_{i,k,m}$ after selection, (potential) conversion, and renormalization by summing over all possible combinations with $M_j A_l B_n$ gametes:

$$\begin{aligned} \bar{w} x^{(cs)}_{i,k,m} = \sum_{j,l,n} \Bigg[& \Big(F^0 B^0_{ij,kl,mn} + (1 - c) F^a B^a_{ij,kl,mn} + (1 - c) F^s B^s_{ij,kl,mn} \Big) x_{i,k,m} x_{j,l,n} \\ & + \frac{1}{4} cF^a B^{a*}_{ij,kl,mn} x_{i,k,m} x_{j,l,n} + \frac{1}{2} cF^a B^{a*}_{ij,kl,nm} x_{i,k,m} x_{j,l,n} \\ & + \frac{1}{4} cF^a B^{a*}_{ij,kl,nm} x_{i,k,n} x_{j,l,m} + \frac{3}{4} cF^s B^s_{ij,kl,mn} x_{i,k,m} x_{j,l,n} + \frac{1}{4} cF^s B^s_{ij,kl,nm} x_{i,k,n} x_{j,l,m} \Bigg]\,. \end{aligned} \quad [16]$$

Some algebra shows that this can be rewritten as

$$\begin{aligned} \bar{w} x^{(cs)}_{i,k,m} = \sum_{j,l,n} \Bigg[& \Big(F^0 B^0_{ij,kl,mn} + F^a \big(1 - \frac{c}{2}\big) B^a_{ij,kl,mn} + F^s \big(1 - \frac{c}{4}\big) B^s_{ij,kl,mn} \Big) x_{i,k,m} x_{j,l,n} \\ & + \frac{c}{4} F^a B^{a*}_{ij,kl,nm} (x_{i,k,m} x_{j,l,n} + x_{i,k,n} x_{j,l,m}) + \frac{c}{4} F^s B^s_{ij,kl,nm} x_{i,k,n} x_{j,l,m} \Bigg]\,. \end{aligned} \quad [17]$$

We assume that recombination among the three loci occurs either before or after conversion (with which it formally commutes), but before selection (which occurs at the end of meiosis because inviable gametes may be produced). Let r_{MA} denote the recombination probability between M and A, and r_{AB} that between A and B. Then recombination between M and A changes $x_{i,k,m}x_{j,l,n}$ to

$$(x_{i,k,m}x_{j,l,n})^{(r_{\mathrm{MA}})} = (1-r_{\mathrm{MA}})x_{i,k,m}x_{j,l,n} + r_{\mathrm{MA}}x_{i,l,n}x_{j,k,m}\,, \tag{18}$$

and recombination among the three loci changes $x_{i,k,m}x_{j,l,n}$ to

$$(x_{i,k,m}x_{j,l,n})^{(r)} = x_{i,k,m}x_{j,l,n} - R\,, \tag{19}$$

where

$$\begin{aligned} R = {} & (1-r_{\mathrm{MA}})r_{\mathrm{AB}}(x_{i,k,m}x_{j,l,n} - x_{i,k,n}x_{j,l,m}) + r_{\mathrm{MA}}(1-r_{\mathrm{AB}})(x_{i,k,m}x_{j,l,n} - x_{i,l,n}x_{j,k,m}) \\ & + r_{\mathrm{MA}}r_{\mathrm{AB}}(x_{i,k,m}x_{j,l,n} - x_{i,l,m}x_{j,k,n})\,. \end{aligned} \tag{20}$$

Therefore, the gamete frequencies after recombination, conversion, and selection are

$$\begin{aligned} \bar{w}x^{(rcs)}_{i,k,m} = \sum_{j,l,n}\Bigg[& \Big(F^0 B^0_{ij,kl,mn} + F^a\big(1-\frac{c}{2}\big)B^a_{ij,kl,mn} + F^s\big(1-\frac{c}{4}\big)B^s_{ij,kl,mn}\Big)(x_{i,k,m}x_{j,l,n})^{(r)} \\ & + \frac{c}{4}F^a B^{a*}_{ij,kl,nm}\Big((x_{i,k,m}x_{j,l,n})^{(r_{\mathrm{MA}})} + (x_{i,k,n}x_{j,l,m})^{(r_{\mathrm{MA}})}\Big) + \frac{c}{4}F^s B^s_{ij,kl,nm}(x_{i,k,n}x_{j,l,m})^{(r)}\Bigg]\,, \end{aligned} \tag{21}$$

where we used $(x_{i,k,m}x_{j,l,n})^{(r)} + (x_{i,k,n}x_{j,l,m})^{(r)} = (x_{i,k,m}x_{j,l,n})^{(r_{\mathrm{MA}})} + (x_{i,k,n}x_{j,l,m})^{(r_{\mathrm{MA}})}$.

If $\rho_s = \rho_a$, so that $F^s = F^a$, then after a brief calculation invoking Eq. (10), we find that Eq. (21) simplifies to

$$\begin{aligned} \bar{w}x^{(rcs)}_{i,k,m} = \sum_{j,l,n}\Bigg[& \Big(F^0 B^0_{ij,kl,mn} + F^a\big(1-\frac{c}{2}\big)\big(1-B^0_{ij,kl,mn}\big)\Big)(x_{i,k,m}x_{j,l,n})^{(r)} \\ & + \frac{c}{4}F^a \bar{B}_{ij,kl,n}\Big((x_{i,k,m}x_{j,l,n})^{(r_{\mathrm{MA}})} + (x_{i,k,n}x_{j,l,m})^{(r_{\mathrm{MA}})}\Big)\Bigg]\,. \end{aligned} \tag{22}$$

The last step in the life cycle is mutation. We allow mutation to occur at loci A and B. We denote the mutation probability from A_k to A_l by $\mu_{A,kl} = \mu_A$, where $k \neq l$, and we define $\mu_{A,kk} = 1-\mu_A$, which is the probability that A_k does not mutate. Analogously, we define $\mu_{B,mn} = \mu_B$ if $m \neq n$, and $\mu_{B,mm} = 1-\mu_B$. Then the frequency of gamete $M_iA_kB_m$ in the next generation is given by

$$x'_{i,k,m} = \sum_{l,n}\mu_{A,lk}\mu_{B,nm}x^{(rcs)}_{i,l,n}\,. \tag{23}$$

For all numerical results shown we assumed $\mu = \mu_A = \mu_B$.

B.1. Dynamics under free recombination. If all three loci are freely recombining, i.e., if $r_{\mathrm{MA}} = r_{\mathrm{AB}} = \frac{1}{2}$, then $(x_{i,k,m}x_{j,l,n})^{(r_{\mathrm{MA}})}$ in Eq. (18) simplifies to

$$(x_{i,k,m}x_{j,l,n})^{(r_{\mathrm{MA}})} = \frac{1}{2}(x_{i,k,m}x_{j,l,n} + x_{i,l,n}x_{j,k,m})\,, \tag{24}$$

and $(x_{i,k,m}x_{j,l,n})^{(r)}$ in Eq. (19) simplifies to

$$(x_{i,k,m}x_{j,l,n})^{(r)} = \frac{1}{4}(x_{i,k,m}x_{j,l,n} + x_{i,k,n}x_{j,l,m} + x_{i,l,n}x_{j,k,m} + x_{i,l,m}x_{j,k,n})\,. \tag{25}$$

Performing these substitutions in Eq. (21), we obtain after a brief computation the representation given in the main text.

C. Allele frequency dynamics. We present the per-generation change of the allele frequencies at the three loci, as caused by recombination, selection, crossover at the target, conversion, and selection. We ignore mutation and assume $\beta = \frac{1}{2}b$.

We denote the allele frequencies of M_1, A_1, and B_1 by $m = \sum_{k,m} x_{1,k,m}$, $p = \sum_{i,m} x_{i,1,m}$, and $q = \sum_{i,k} x_{i,k,1}$, respectively. We denote the pairwise linkage disequilibria between loci A and B, M and A, and M and B by D_{AB}, D_{MA}, and D_{MB}, respectively. The three-way linkage disequilibrium is D_{MAB}. These linkage disequilibria are defined as follows (e.g. 3):

$$D_{\mathrm{AB}} = \sum_i x_{i,1,1}\sum_i x_{i,2,2} - \sum_i x_{i,1,2}\sum_i x_{i,2,1}\,, \tag{26a}$$

$$D_{\mathrm{MA}} = \sum_m x_{1,1,n}\sum_n x_{0,2,n} - \sum_n x_{1,2,n}\sum_n x_{0,1,n}\,, \tag{26b}$$

$$D_{\mathrm{MB}} = \sum_k x_{1,k,1}\sum_k x_{0,k,2} - \sum_k x_{1,k,2}\sum_k x_{0,k,1}\,, \tag{26c}$$

$$D_{\mathrm{MAB}} = x_{1,1,1} - mpq - mD_{\mathrm{AB}} - pD_{\mathrm{MB}} - qD_{\mathrm{MA}}\,. \tag{26d}$$

We note that $D_{\mathrm{AB}} > 0$ if A_1 and B_1 are associated, $D_{\mathrm{MA}} > 0$ if M_1 and A_1 are associated, $D_{\mathrm{MB}} > 0$ if M_1 and B_1 are associated, and $D_{\mathrm{MAB}} > 0$ if M_1 is associated with the gamete A_1B_1.

With the help of *Mathematica* (see the Supplementary *Mathematica* notebook) the per-generation change of the allele frequencies m, p and q, as well as of the four linkage disequilibria can be computed from the gamete-frequency changes given in Eq. (21). Below we focus on the allele-frequency dynamics, especially on the frequency m of the specific modifier allele M_1. The respective recursion equations are formidable, and those for the linkage disequilibria are unwieldy. Therefore, the latter are only given in the Supplementary *Mathematica* notebook.

Before presenting the relevant equations, we recall that according to a classical result by (4), the allele-frequency change at a single diallelic locus under viability selection can be written as $\bar{w}\Delta p = \frac{p(1-p)}{2}\frac{d\bar{w}}{dp}$. This can be extended to multiple loci, but becomes much more complicated because then $\bar{w}$ depends on the allele frequencies at the involved loci and the linkage disequilibria among them, generated for instance by epistasis (e.g. 5). In the present model, even in the absence of recombination among the loci M, A and B, the change in gamete frequencies cannot be represented with the help of a quadratic form because our model includes conversion, which causes asymmetries and generates recombination-like terms. In particular, there is no matrix W such that the change Δx in the vector x of gamete frequencies can be represented as $\bar{w}\Delta x = (Wx) \circ x$ (see 1, 2), who treated models with only the loci A and B). Nevertheless, below we will separate the terms resulting from direct selection on the three loci from the other terms because their contribution to the per generation response is most substantial.

In order to write the equations for the allele frequencies in compact form, it is convenient to use the following compound parameters:

$$\varphi = \frac{bf}{2}\rho_a\,, \qquad [27a]$$

$$\gamma = \frac{bc}{4}\Big(1 - f(1-\rho_a)\Big)\,, \qquad [27b]$$

$$\rho_\Delta = \frac{b^2 f}{4}(\rho_s - \rho_a)\,. \qquad [27c]$$

We note that $\varphi = \frac{1}{2}b(F^a - F^0)$ and $\rho_\Delta = \frac{1}{4}b^2(F^s - F^a)$.

Because many of the terms that have a large effect on the allele-frequency dynamics derive from mean fitness $\bar{w}$, we present it explicitly:

$$\begin{aligned}
\bar{w} &= 1 - f + \varphi + \varphi m(2p-1)(2q-1) && [28a]\\
&\quad + \varphi\Big(2mD_{\mathrm{AB}} + (2q-1)D_{\mathrm{MA}} + (2p-1)D_{\mathrm{MB}} + D_{\mathrm{MAB}}\Big) && [28b]\\
&\quad + \rho_\Delta\Big[(1-m) + 2m\Big(p^2(2q-1)^2 + 2(1-q)^2 + p(2q-1)(3-2q)\Big)\Big] && [28c]\\
&\quad + \rho_\Delta\Big[4D_{\mathrm{AB}}\Big(2m\big(pq + (1-p)(1-q) + D_{\mathrm{AB}}\big) + (2q-1)D_{\mathrm{MA}} + (2p-1)D_{\mathrm{MB}} + D_{\mathrm{MAB}}\Big) && [28d]\\
&\qquad + D_{\mathrm{MA}}(2q-1)\big(2 + (2p-1)(2q-1)\big) + 4D_{\mathrm{MB}}\big((1-q)(2p-1) + p^2(2q-1)\big) && [28e]\\
&\qquad + 4D_{\mathrm{MAB}}\big(pq + (1-p)(1-q) + D_{\mathrm{AB}}\big)\Big]\,. && [28f]
\end{aligned}$$

We observe that the terms in eqs. Eq. (28c) – Eq. (28f) occur only if $F^s > F^a$, or $\rho_s > \rho_a$. If linkage disequilibria can be ignored, only the terms in Eq. (28a) and Eq. (28c) are relevant. Clearly, the mean fitness does not depend on the probability of conversion.

We note that when the unspecific modifier M_0 is fixed ($m = 0$), then the mean fitness is $\bar{w} = 1 - f + \varphi + \rho_\Delta$, which is independent of the allele frequencies p and q. If the specific modifier M_1 is fixed ($m = 1$), then mean fitness in a hot state ($p = q = 1$ or $p = q = 0$) is $\bar{w} = 1 - f + 2\varphi + 4\rho_\Delta$, and in a cold state ($p = 1, q = 0$ or $p = 0, q = 1$) it is $\bar{w} = 1 - f$.

C.1. Change of the frequency m of the specific modifier allele M_1. Having Wright's classcial result in mind, we first calculate $\frac{\partial \bar{w}}{\partial m}$ to obtain the contribution to the allele-frequency change resulting from direct selection on the modifier locus. We find

$$\begin{aligned}
\frac{\partial \bar{w}}{\partial m} &= \varphi\Big((2p-1)(2q-1) + 2D_{\mathrm{AB}}\Big) && [29a]\\
&\quad + \rho_\Delta\Big(\big[(3-2q)(2p-1) + 2p^2(2q-1)\big](2q-1) && [29b]\\
&\qquad + 8\big(pq + (1-p)(1-q)\big)D_{\mathrm{AB}} + 8D_{\mathrm{AB}}^2\Big)\,. && [29c]
\end{aligned}$$

The dependence of $\frac{\partial \bar{w}}{\partial m}$ on p and q indicates the direct frequency-dependent effects of the A and the B locus on the modifier. The dependence on D_{AB} indicates the influence of these loci through the linkage disequilibrium caused by epistasis.

The term $\big[(3-2q)(2p-1) + 2p^2(2q-1)\big](2q-1)$ in eq. Eq. (29b) can be positive or negative. The region of (p, q) values in which this term is positive is larger than where it is negative; and it is more strongly positive than negative (see Supplementary *Mathematica* notebook). Thus, averaged over all (p, q) combinations, this term favors the specific modifier M_1 if $\rho_s > \rho_a$. Moreover, $pq + (1-p)(1-q) \geq 0$ holds always.

In general, and in particular because we assume free recombination, the linkage disequilibrium term D_{AB} is small and can often be neglected. Exceptions occur when both p and q are close to $\frac{1}{2}$. We will discuss this below in connection with the figures.

By re-parameterization and after considerable algebra we obtain from Eq. (21) the response of the allele frequency m:

$$\bar{w}\Delta^{(rcs)}m = \frac{m(1-m)}{2}\frac{\partial \bar{w}}{\partial m} \quad [30a]$$
$$+ \frac{1}{2}\varphi\Big[(2q-1)D_{\mathrm{MA}} + (2p-1)D_{\mathrm{MB}} + D_{\mathrm{MAB}}\Big] \quad [30b]$$
$$+ 2\rho_\Delta\left[\frac{1}{4}(2q-1)\big(2+(2p-1)(2q-1)\big)D_{\mathrm{MA}} + \big(pq+(1-p)(1-q)\big)D_{\mathrm{MAB}}\right] \quad [30c]$$
$$+ T_m\,, \quad [30d]$$

where

$$T_m = \varphi D_{\mathrm{MA}}D_{\mathrm{MB}} + \rho_\Delta\bigg((2q-1)^2D_{\mathrm{MA}}^2 + 4\big(pq+(1-p)(1-q)\big)D_{\mathrm{MA}}D_{\mathrm{MB}} + 2\big(p^2+(1-p)^2\big)\big)D_{\mathrm{MB}}^2 + 2D_{\mathrm{AB}}\big[(2q-1)D_{\mathrm{MA}} + (2p-1)D_{\mathrm{MB}}\big] + 4D_{\mathrm{MAB}}\big[D_{\mathrm{AB}} + (2q-1)D_{\mathrm{MA}} + (2p-1)D_{\mathrm{MB}}\big] + 4D_{\mathrm{MAB}}^2\bigg) \quad [31]$$

contains only products of linkage disequilibria, which are of smaller order than the other terms. The influence of the term T_m on the dynamics is negligible. In general, also the three-way linkage disequilibrium D_{MAB} is extremely small and can be neglected unless all of m, p and q are close to $\frac{1}{2}$ (see figures below). The reader may note that terms invoking ρ_Δ occur only if symmetric binding leads to higher fitness than asymmetric binding. Finally, we note that neither $\frac{\partial \bar{w}}{\partial m}$ nor any of the expressions in eqs. Eq. (30b) – Eq. (30d) contains terms with m. Therefore, there is indeed no dominance effect of M_0M_1, i.e., selection strength on heterozygotes is exactly intermediate between those on homozygotes.

We performed extensive numerical iterations of the full recursion equations. Representative and particularly interesting examples of the evolutionary dynamics are shown in the figures below. These and many more simulations have shown that the single-generation change Δm of M_1 can be closely approximated by

$$\bar{w}\Delta m \approx \frac{bf}{4}m(1-m)T\,, \quad [32a]$$

where

$$T = \big(\rho_a + b(\rho_s-\rho_a)\big)(2p-1)(2q-1) \quad [32b]$$
$$+ \tfrac{1}{2}b(\rho_s-\rho_a)\big((1-p)^2+p^2\big)(2q-1)^2 \quad [32c]$$
$$+ 2\big[\rho_a + 2b(\rho_s-\rho_a)\big((1-p)(1-q)+pq\big)\big]D_{\mathrm{AB}} \quad [32d]$$

is a frequency dependent term.

The linkage disequilibrium term in Eq. (32d) is more relevant when both p and q are close to $\frac{1}{2}$ (for an example, see Fig. S2.D). When p and q oscillate (as is the case when the dynamics is PRDM9 driven and leads to hotspot/coldspot oscillations), then D_{AB} is negative, very small (because of the general estimate $-\min\{pq,(1-p)(1-q)\} \le D_{\mathrm{AB}}$ and because at least one of the allele frequencies is very small). The term in Eq. (32b) leads to oscillations in the modifier frequency (provided the modifier is polymorphic), driven by oscillations of p and q. This term is positive near hotspots and negative near coldspots. The term in Eq. (32c) does not lead to oscillations. It is only relevant when symmetric bindings result in crossovers more often than asymmetric do, i.e., if $\rho_s > \rho_a$. Then it is positive and shows that the overall effect of symmetric binding is to favor the specific modifier M_1.

The representation in Eq. (32) shows that, in contrast to some classical models of modifier evolution (in which the modifier is only under indirect selection through linkage disequilibrium or the strength of direct selection is on the order of the mutation rate), in the present model the modifier is under direct selection, which is frequency dependent and generally strong (see, in particular, Sect. C.4 below).

C.2. Change of the frequency p of the targeting allele A_1. The single-generation change Δp of the frequency of allele A_1 can be closely approximated by

$$\bar{w}\Delta p \approx \frac{bf}{2}p(1-p)m(2q-1)\left[\rho_a + \frac{b}{2}(\rho_s-\rho_a)\Big(2+(2p-1)(2q-1)\Big)\right]. \quad [33]$$

Obviously, selection on the targeting locus A ceases if the unspecific modifier is frequent (m is small), and it is maximized if M_1 is fixed. If $m>0$, then $\Delta p > 0$ if $q > \frac{1}{2}$, and $\Delta p < 0$ if $q < \frac{1}{2}$. More specifically, we obtain

$$\bar{w}\Delta p \approx \frac{bf}{2}p(1-p)m \times \begin{cases} \big[\rho_a + \frac{b}{2}(\rho_s-\rho_a)(2p+1)\big] > 0 & \text{if } q\approx 1\,, \\ \big[-\rho_a - \frac{b}{2}(\rho_s-\rho_a)(3-2p)\big] < 0 & \text{if } q \approx 0\,. \end{cases} \quad [34]$$

In the first case, if initially A_1 is rare (as in the vicinity of the coldspot A_2B_1, by which we mean $p \approx 0$ and $q \approx 1$), then A_1 will (rapidly) rise to high frequency and the system will approach the hotspot A_1B_1 (i.e., $p \approx q \approx 1$), provided M_1 is frequent. In the second case, and again provided M_1 is frequent, if initially A_2 is rare (as in the vicinity of the coldspot A_1B_2), then A_2 will (rapidly) rise to high frequency and the system will approach the hotspot A_2B_2. The effect of symmetric binding ($\rho_s > \rho_a$) is to increase selection strength on A near hotspots, and to weaken it near coldspots. Furthermore, eq. Eq. (33) shows that if $\rho_s = \rho_a$, then there is no dominance, i.e., selection strength on heterozygotes is exactly intermediate between those of homozygotes. If $\rho_s > \rho_a$, then a component of partial dominance is added by the term $(2p-1)(2q-1)$.

We note that although D_{AB} plays essentially no role in the evolution of p and q when p and q oscillate (because then at least one of them is very close to the boundary of the state space), D_{AB} is relevant for the long-term evolution if both allele frequencies are far away from their extreme values. This is qualitatively similar to the model treated in Úbeda et al. (2023). In the present model, in which the focus is on the evolutionary stability of non-PRDM9-guided vs. PRDM9-guided recombination hotspots, the main interest is in situations (parameters regions) in which stable oscillations do (or can) occur if the modifier M_1 is present.

C.3. Change of the frequency q of the targeting allele B_1. The single-generation change Δq of the frequency of allele B_1 can be closely approximated by

$$\bar{w}\Delta q \approx \frac{bf}{4} q(1-q)m\left(\left[2\rho_a + 2b(\rho_s-\rho_a) - \frac{c}{f}\big(1-f(1-\rho_a)\big)\right](2p-1) + 2b(\rho_s-\rho_a)(2q-1)\big(p^2+(1-p)^2\big)\right). \qquad [35]$$

This equation also shows that there is no dominance at locus B.

As in the case of selection on locus A, selection on the target B ceases if the unspecific modifier is frequent. By simple algebra we obtain

$$\bar{w}\Delta q \approx \frac{bf}{4} q(1-q)m \times \begin{cases} \big[c(1-\rho_a) + 2\rho_a + 4b(\rho_s-\rho_a)q - c/f\big] & \text{if } p \approx 1\,, \\ \big[-c(1-\rho_a) - 2\rho_a - 4b(\rho_s-\rho_a)(1-q) + c/f\big] & \text{if } p \approx 0\,. \end{cases} \qquad [36]$$

If we define

$$f_{\mathrm{cyc}} = \frac{c}{c(1-\rho_a) + 2\rho_a + 4b(\rho_s-\rho_a)}\,, \qquad [37]$$

then $c(1-\rho_a) + 2\rho_a + 4b(\rho_s-\rho_a) - c/f = c(1/f_{\mathrm{cyc}} - 1/f)$. Therefore, we conclude from Eq. (36) that the following statements hold if and only if $0 < f < f_{\mathrm{cyc}}$: $\Delta q < 0$ for every $0 < q < 1$ if $p \approx 1$, and $\Delta q > 0$ for every $0 < q < 1$ if $p \approx 0$. Thus, if $f < f_{\mathrm{cyc}}$ then conversion is strong enough relative to fecundity selection to drive the hot allele at the target to extinction.

Together with the insight from Eq. (34) that $\Delta p > 0$ if $q \approx 1$, and $\Delta p < 0$ if $q \approx 0$, we conclude that near the boundary of the (p,q) state space and if the specific modifier is sufficiently frequent, selection drives transitions along the path $(p,q) = (0,0) \longrightarrow (0,1) \longrightarrow (1,1) \longrightarrow (1,0) \longrightarrow (0,0)$, i.e., from hotspot to coldspot, from coldspot to hotspot, and so on. We remark that if $f > f_{\mathrm{cyc}}$, then the hotspot equilibria are locally stable and apparently attract all trajectories. Thus, they are permanent recombination hotspots, and M_1 will be evolutionarily stable. Therefore, in this study we focus on the parameter range $f < f_{\mathrm{cyc}}$ because only then Red-Queen dynamics can occur.

In Section C.5 we provide graphical illustrations of several interesting cases of modifier evolution, which complement the central results in the main text.

C.4. Strength of selection. The full equations Eq. (30) for Δm show that conversion (whose strength is c) does not directly affect modifier selection. The effects of conversion act only indirectly through their direct effects on the allele frequency q (eq. Eq. (35)) and the linkage disequilibria (see *Mathematica* notebook).

From Eq. (32) and Eq. (33) we infer immediately that there is no direct selection on m and p if $q = \frac{1}{2}$. However, in the vicinity of hotspots or coldspots, the strength of selection on M_1 is of similar magnitude as that on A_1 provided M_1 is close to fixation ($m \approx 1$). By simple calculations we obtain from Eq. (32) the selection coefficients at the modifier locus M near hotspots and coldspots:

$$\frac{\bar{w}\Delta m}{m(1-m)} \approx \frac{bf}{4} \times \begin{cases} \big(\rho_a + \frac{3b}{2}(\rho_s-\rho_a)\big) & \text{if } p \approx 1 \text{ and } q \approx 1\,, \\ \big(\rho_a + \frac{3b}{2}(\rho_s-\rho_a)\big) & \text{if } p \approx 0 \text{ and } q \approx 0\,, \\ \big(-\rho_a - \frac{b}{2}(\rho_s-\rho_a)\big) & \text{if } p \approx 1 \text{ and } q \approx 0\,, \\ \big(-\rho_a - \frac{b}{2}(\rho_s-\rho_a)\big) & \text{if } p \approx 0 \text{ and } q \approx 1\,. \end{cases} \qquad [38]$$

From Eq. (33) we obtain the corresponding selection coefficients at the target locus A:

$$\frac{\bar{w}\Delta p}{p(1-p)} \approx \frac{bf}{2} m \times \begin{cases} \big(\rho_a + \frac{3b}{2}(\rho_s-\rho_a)\big) & \text{if } p \approx 1 \text{ and } q \approx 1\,, \\ \big(-\rho_a - \frac{3b}{2}(\rho_s-\rho_a)\big) & \text{if } p \approx 0 \text{ and } q \approx 0\,, \\ \big(-\rho_a - \frac{b}{2}(\rho_s-\rho_a)\big) & \text{if } p \approx 1 \text{ and } q \approx 0\,, \\ \big(\rho_a + \frac{b}{2}(\rho_s-\rho_a)\big) & \text{if } p \approx 0 \text{ and } q \approx 1\,. \end{cases} \qquad [39]$$

Comparison shows that in the vicinity of hotspots and of coldspots the strength of selection on M_1 is about half of that as on A_1 (provided M_1 is nearly fixed), only their signs are opposite if $p \approx 0$ because A_2 is favored if B_2 is common. In addition, Eq. (38) informs us that the increase in selection intensity in favor of M_1 near hotspots caused by a fitness advantage of symmetric binding ($\rho_s > \rho_a$) exceeds its induced decrease near coldspots.

Whether selection at locus B is weaker or stronger than at locus A depends on the relation between f and c. If f is much less than f_{cyc}, then selection at B is stronger.

C.5. Illustration of the evolution of allele frequencies, linkage disequilibria, and mean fitness. In Figures S2 – S6 we display the time dependence of the allele frequencies (top panel), of the linkage disequilibria (middle panel), and of the mean fitness for selected cases occurring in Fig. 6 in the main text. In addition, we illustrate the effects of a higher mutation rate ($\mu = 10^{-7}$). These figures complement the systematic results concerning transitions between types of hotspots presented in Figures 5 – 7 in the main text. The following values of f_{cyc} explain the upper bounds of the range of values f considered in Figs. 5–7 in the main text. If $\rho_s = \rho_a = 0.75$ (as in Fig. 5), then $f_{\text{cyc}} \approx 0.571$. If $(\rho_a, \rho_s) = (0.74, 0.76)$, $(\rho_a, \rho_s) = (0.7, 0.8)$, $(\rho_a, \rho_s) = (0.6, 0.9)$, and $(\rho_a, \rho_s) = (0.5, 1)$ (as in Fig. 6), then $f_{\text{cyc}} \approx 0.549$, 0.476, 0.357, and 0.286, respectively. In all these figures, $b = c = 1$.

All results were obtained by numerical iteration of the full recursion equations for the gamete frequencies, Eq. (21) and Eq. (23), which were then transformed to allele frequencies and linkage disequilibria. We recall that for all numerical results we assume free recombination among all three loci, i.e., $r_{MA} = r_{MB} = r_{AB} = \frac{1}{2}$.

In Fig. S2, we show cases where invasion of a rare unspecific modifier allele M_0 leads to replacement of the resident specific modifier allele M_1. In this case, the fitness advantage of symmetric binding is tiny ($\rho_a = 0.74$, $\rho_s = 0.76$). Very similar graphs are obtained if symmetric binding has no advantage ($\rho_a = \rho_s = 0.75$; results not shown, but compare the relevant panels of Figs. 5 and 6 in the main text). This figure also illustrates that in this range of parameters the main effect of a higher mutation rate is to slow down the replacement of the specific modifier.

In Fig. S4, the fitness advantage of symmetric binding is somewhat increased ($\rho_a = 0.7$, $\rho_s = 0.8$). This does not lead to a qualitative change in the observed patterns compared to Fig. S2 if $\mu = 10^{-9}$ (results not shown), however, it does if $\mu = 10^{-7}$. In this case, the unspecific modifier can invade, but not replace the specific one. Instead, both modifier and allele-frequency dynamics become cyclical and both modifiers coexist in an oscillatory manner. The left panels show the first 5 000 generations, the right panels show generations 95 000 – 100 000, when the dynamics has approached stationary cycling.

In Figs. S5 and S6, the fitness advantage of symmetric binding is further increased ($\rho_a = 0.6$, $\rho_s = 0.9$). Figure S5 demonstrates that cycling of the two modifier alleles also occurs for very small mutations rates ($\mu = 10^{-9}$). Here, a particularly interesting case is shown in which the allele-frequency dynamics at all three loci is cyclical, apparently with a period of four. Accordingly, (some of the) linkage disequilibria oscillate as does the mean fitness (which is determined by the realized crossover rate near the target).

The left panels in Fig. S6 show that for the same parameters as in Fig. S5, but the higher mutation rate $\mu = 10^{-7}$, the unspecific modifier cannot become established. It can invade initially but, unless it is very frequent, in the long run it is out-competed by the specific modifier. Thus, a higher mutation rate makes the specific modifier more resistant against replacement. The right panels show that for the low mutation rate $\mu = 10^{-9}$, this dynamics occurs only for smaller values of f. In addition, a more frequent modifier M_0 will replace the specific M_1 under a wide variety of initial conditions (cf. Fig. 6 in the main text).

We emphasize that Figures S4 and S5 illustrate a novel evolutionary scenario, in which stable coexistence of both modifier alleles occurs by permanent oscillations in modifier allele frequencies and concurrent complex oscillations in frequencies of targeting and target alleles. These differ from the previously reported (1, 2) Red-Queen-like regular oscillations at targeting and target locus that occur when the specific (PRDM9-like) modifier is fixed. We note that for parameter combinations that admit coexistence of both modifier alleles, it depends strongly on the initial conditions whether such stable coexistence can be established. For other initial conditions, invasion of a rare M_1 (or M_0) does not occur, and for still others one allele can replace the other (results not shown).

In Fig. S7 the exact, scaled per-generation response $\bar{w}\Delta m$ (red curves) of the modifier allele M_1 is compared with the approximation given in Eq. (32) (black dashed curves), where additionally D_{AB} is set to 0, and with the very simple approximation $\rho_a m(1-m)(1-2p)(1-2q)$ (blue dashed curves), in which the additional contribution of symmetric binding $(\rho_s - \rho_a)$ is ignored. The figures show that, generally, the approximation Eq. (32) fits the true response very accurately, despite the neglect of D_{AB}. The additional contribution by symmetric binding cannot be ignored unless $(\rho_s - \rho_a)$ is very small. However, it is important to keep in mind that Eq. (32) predicts the per-generation response on the basis of the exact values of the allele frequencies and the linkage disequilibria in the current generation. Some of the linkage-disequilibrium terms occurring in the full recursions cannot be neglected without changing the long-term dynamics, in some cases even qualitatively (results not shown).

We note that if $m = 0$ and $\mu = 0$, then all allele frequencies remain constant (because then also the linkage disequilibria with locus M vanish and the mean fitness is independent of the allele frequencies). If, initially, $D_{\text{AB}} \neq 0$, then D_{AB} converges to 0 (see supplementary *Mathematica* notebook). If $m = 0$ and $\mu > 0$, then the allele frequencies p and q converge to 0.5, but on the long time scale $1/\mu$. In the figures shown, we always chose $b = c = 1$ because this speeds up the simulations. Smaller values of b and c lead to qualitatively similar behavior, but on a longer time scale (results not shown). Also the range of values f in which analogous dynamics occur may differ. This can be seen from the expression f_{cyc} in Eq. (37) for the upper bound of the range in which Red Queen dynamics does occur and from the numerical examples presented here. In particular, numerical

iteration shows that the length of the cycles in the hotspot dynamics increases considerably with decreasing b or c. Also, in accordance with intuition, decreasing mutation rates increase the cycle length, but not dramatically.

C.6. Evolution with multiple alleles and target sites. Our model assumes that only two alleles segregate at the targeting locus. In nature, however, the PRDM9 locus is known to be highly polymorphic. In addition, our model assumes that there is only one target site. In nature, however, the number of potential targets for a PRDM9 protein targeting a specific motif is high (presumably, in the thousands; e.g., (6–9)). Here, we discuss why nevertheless we expect that our model yields insights for the evolution of PRDM9-guided recombination hotspots when there are multiple targets and target sites.

(i) Implications of having multiple alleles at PRDM9 Although we model two alleles segregating at a target locus the insight gained can apply to multiple alleles. For simplicity, the targeting-target co-evolutionary model we build upon assumes the existence of two alleles at each locus and forward and backward mutation between the alleles at each locus. This simplification allows the derivation of analytical results and an in-depth understanding of the dynamics. Assuming forward and backward mutation results in cycles where the same combination of targeting and target alleles repeats, that is $A_1B_2 \to A_2B_2 \to A_2B_1 \to A_1B_1 \to A_1B_2 \to \ldots$ (2). From a phenotypic perspective the recurrence of targeting and target alleles translates into a recombination hotspot in the same genomic location oscillating between hot and cold phases over evolutionary time (2). In nature however, the zinc finger domain in PRDM9 —domain that determines the matched sequence— consists of a sequence of 10 nucleotides with 4 nucleotides to choose from in each position (10, 11). Because mutation rates are low, in most cases a mutant target motif will differ from its original sequence in one base. A second mutation will result in reversion to the ancestral sequence with probability $\frac{1}{30}$ and acquisition of a new derived state with probability $\frac{1}{30}$. Forward mutations are therefore 29-times more likely than backward mutations, but our analytical model still applies under the assumption of forward mutations.

Our model assumes that subscripts of alleles A and B represent specific nucleotide sequences. However, they are only labels and the results of the model apply when replacing old labels with new labels. Assuming that mutations are no longer backward but only forward (μ_A and μ_B denote the probabilities of mutation $A_1 \to A_2 \to A_3 \ldots$ and $B_1 \to B_2 \to B_3 \ldots$), we obtain cycling dynamics where new combinations of targeting and target alleles succeed old ones, that is $A_1B_2 \to A_2B_2 \to A_2B_3 \to A_3B_3 \to A_3B_4 \to \ldots$. From a phenotypic perspective the non-recurrence of targeting and target alleles translates into recombination hotspots in new genomic locations becoming hot as the ones in old locations become cold. What is relevant about these subindices is whether the sequences match or do not match. In a population of mismatching targeting and target sequences, fertility selection favours fixation of a matching targeting sequence. When the previous allele is fixed, biased gene conversion drives fixation of a mismatching target sequence. Co-evolution results in an alternation between mismatching and matching haplotypes that is maintained over evolutionary time. This is true independently of the labels used to denote matching or mismatching alleles. We thus contend that the model we choose to build upon captures the key-features of the co-evolution between target and targeting loci without sacrificing realism in terms of non-recurrence of sequences and location of hotspots. This conclusion has been validated by repeated extensions of the one target model to multiple targets (1, 2, 12).

(ii) Implications of having multiple target sites In the presence of multiple target sites, PRDM9 alleles can build genetic associations (linkage disequilibrium) with each of its target loci. When dynamics are oscillatory there is a small increment in the association between mismatching targeting and target loci that results in a lower crossover rate. This small association may be magnified by the multiplicity of target sits thus enhancing selection for unspecific modifiers.

C.7. Evolution in finite populations. Our model considers an infinite population and as such ignores random genetic drift. Because the oscillations in allele frequencies observed often lead to frequencies very close to 0 or 1, elimination of such rare alleles will occur even in large populations. Here, we discuss why nevertheless we expect that our model yields important insights for the evolution of PRDM9-guided recombination hotspots in finite populations.

(i) Implications for the co-evolution of targeting and target loci We start by discussing the evolutionary dynamics at the targeting and target loci A and B if alleles at A are specific —if they are unspecific, selection at A and B is absent because then binding probabilities are independent of the genotypes at A and B. During stable oscillations at the loci A and B, the minimum allele frequencies are typically on the order of μ to 10μ. These extreme allele frequencies occur during the short period when a transition from a hotspot or coldspot to the opposite type occurs, i.e., during the evolution along the four edges of the (p, q) state space. During these transitions, there is a significant or even high probability that the rare allele will be lost by random genetic drift (unless $N\mu$ is much larger than 1). However, we note that the time spent at extremely low frequencies (10^{-6} or lower) is much shorter than it appears in the figures because at this scale of resolution frequencies below 10^{-3} cannot be resolved. If, for instance, such a loss occurs during the transition from the vicinity of the A_1B_1 hotspot towards the A_1B_2 coldspot, it will be the A_2 allele that is lost and, in the (likely) absence of further mutation during this short period, A_1B_2 will become fixed in a finite population (indeed, eq. Eq. (35) shows that selection strength on B_2 is maximized if A_2 is absent). As soon as a suitable mutant is available at some target (now re-labeled to B) together with a matching allele at locus A, selection kicks in and a hotspot at the new target evolves, as described by our deterministic model. This process will lead to a rapid succession of hotspots, and thus to an elevated recombination rate at this chromosome, if the number of potential targets is large and selection is sufficiently strong. The reason is that the waiting time for the emergence of new hotspots will be inversely proportional to the selective advantage of s of potential mutants because, according to Haldane's rule, an advantageous mutant has a fixation probability of only about $2s$. Once a suitable mutant has reached a certain frequency to avoid immediate loss,

selection can pick it up and quickly drive it to high frequency by the mechanism described in our model. Thus, in contrast to the deterministic model, coldspot-hotspot transitions will be irregular with a stochastic waiting time between them.

In summary, in finite populations there will be fewer oscillations at a given single target. However, alternative targets will play a similar role as in our deterministic model, with hotspots popping up at different locations and disappearing again. For a simpler two-locus model (without an advantage of symmetric binding and without a modifier, i.e., the targeting alleles at A were fully specific), the applicability of this argument was shown using individual based simulations with 10 and 100 potential targets (see 2, 12).

(ii) Implications for modifier evolution The above arguments are relevant only when considering invasion of M_0 in a resident M_1 population (because then oscillations are in progress), or when M_1 can invade a resident M_0 population (and oscillations will be initiated). In the vast majority of cases when M_1 does not become established after emerging in a resident M_0 population, the loss occurs quickly in the deterministic model and without oscillations. In this case, essentially the same pattern will occur in a finite population.

Because selection on the modifier locus is about half as strong as selection on the targeting locus A (see Sect. C.4), random genetic drift will have a very limited influence on modifier evolution unless a modifier allele gets very rare during the oscillations. Importantly, if a targeting or target allele gets fixed by random drift during a transition from one type of hotspot to the other, the selection strength on the modifier is in fact intensified and maximized in favor of M_1 near hotspots, and maximized in favor of M_0 near coldspots. Thus, modifier evolution proceeds during periods of reduced or no variability at the primary loci. Moreover, the structure of the deterministic equation for Δm remains unchanged after potential relabeling of alleles at A or of relabeling the target site B. The parameters, especially the binding probability b, may change however.

We expect that the influence of random drift on modifier evolution is limited in large populations because our deterministic results show that oscillations in m (if they occur) are much less extreme than those at loci A and B (well visible in some of the figures and confirmed by additional logarithmic plots). Of course, fixation of a modifier allele can occur faster than suggested by the deterministic model (in which, strictly speaking, fixation never occurs). In some cases, the modifier M_0 will invade but may be lost during the initial fluctuations (cf. Fig. 4.A in the main text). We also note that oscillations in m are about twice as fast as in p and q, which confirms that selection on M is strong. This factor of two is not surprising because M_1 is favored near hotspots independently of their genotype (A_1B_1 or A_2B_2), and M_0 is favored near the coldspots.

In summary, in a large finite population we expect similar patterns of modifier evolution as in the deterministic case. The pattern of oscillations will be more irregular and fixation or loss can occur faster. Hotspot locations will change over sufficiently long evolutionary times, and the parameter regions in which either one or the other modifier is evolutionarily stable, or both are simultaneously evolutionarily stable, or both coexist for a long time, will depend on the underlying parameter values. The investigation of a finite population model would be an interesting and challenging topic for future research. Our present, deterministic model is designed to provide fundamental theoretical insights into the potential evolutionary origin and the maintenance of PRDM9-guided recombination hotspots.

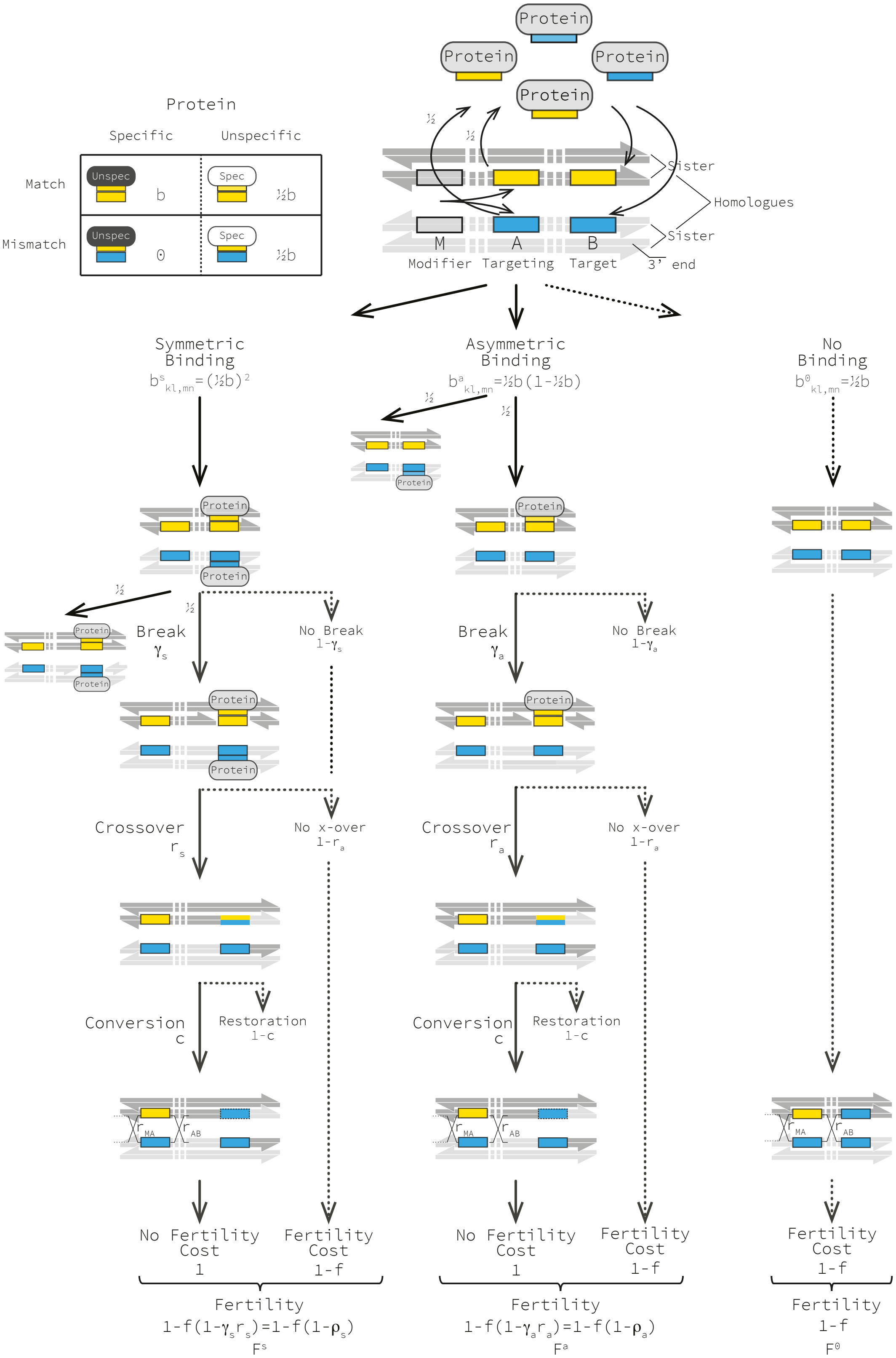

Fig. S1. Sketch of the model. A targeting allele produces a pool of proteins that attempt to bind motifs at a target locus. Homozygotes for the unspecific allele produce unspecific proteins. Unspecific proteins bind any target allele with probability $\frac{1}{2}b$. Homozygotes for the specific allele produce specific proteins. Specific proteins bind matching alleles (represented in the figure by the same colour of targeting and target alleles) with probability b but they do not bind mismatching alleles. Binding attempts can result in either symmetric binding, asymmetric binding, or no binding (figure presents the case of heterozygous targeting and target loci). Following symmetric or asymmetric binding events double-strand breaks take place with probabilities γ_s and γ_a respectively. Recombination results in crossover between alleles at each of the three loci with probability one half. Recombination also results in crossover between flanking regions of the target locus with probabilities r_s or r_a if the double-strand break was initiated by symmetric or asymmetric binding, respectively. (Note that only the compound parameters $\rho_s = \gamma_s r_s$ and $\rho_a = \gamma_a r_a$ in Eq. (12) enter the dynamical equations.) Crossover results in conversion of the mismatched sequence with probability c. Alleles segregate into gametes fairly. If there is no crossover at the target locus, alleles may not segregate properly resulting in non-viable gametes with probability f.

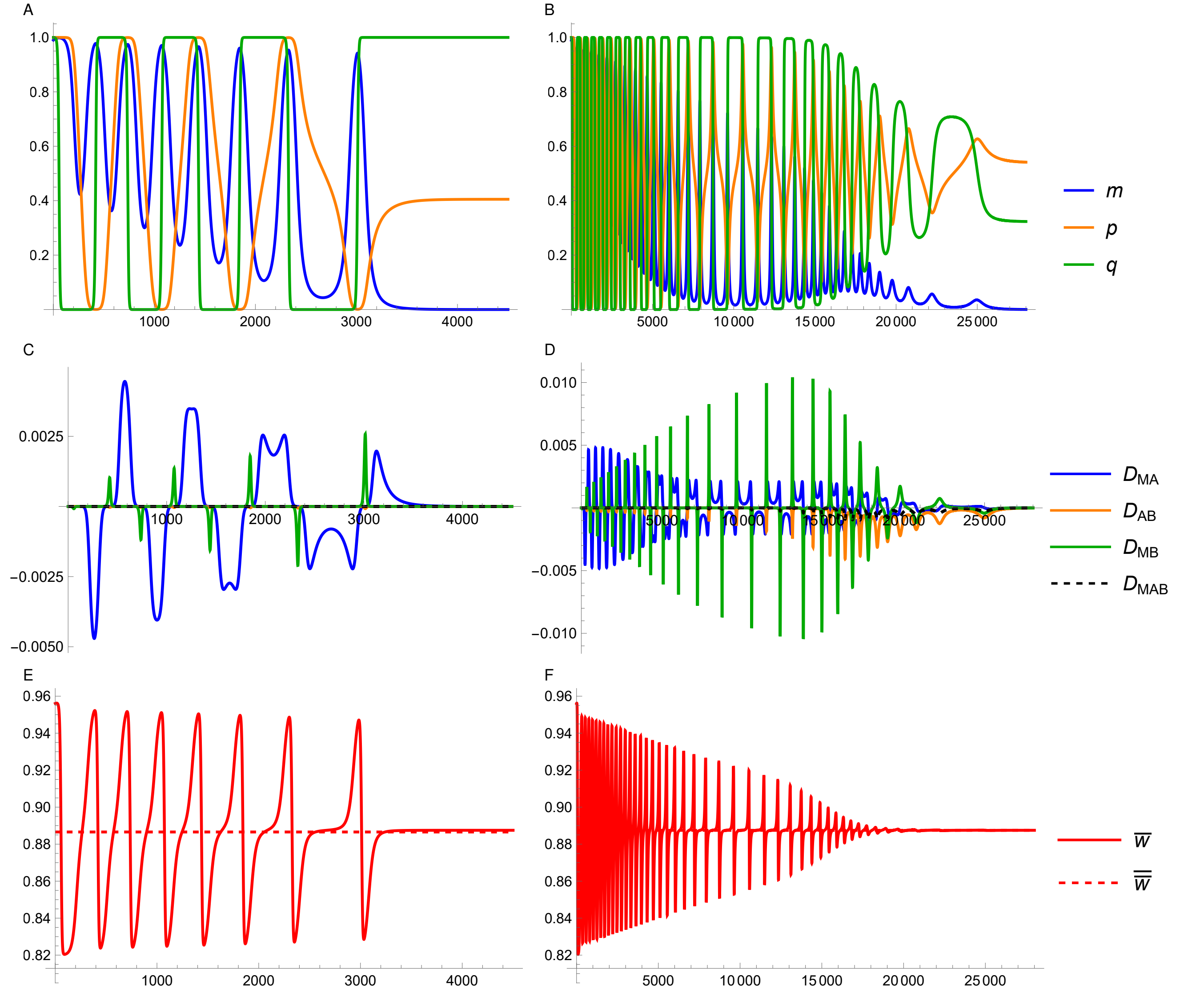

Fig. S2. Invasion of a rare unspecific modifier allele M_0 and eventual replacement of the resident specific allele M_1. This leads to the exclusive use of non-PRDM9 hotspots. Panels A and B show the trajectories of the allele frequencies m, p, and q of M_1, A_1, and B_1. Panels C and D show the four linkage disequilibria. Panels E and F show the time-dependent mean fitness $\bar{w}$ and the mean fitness averaged over all generations, $\bar{\bar{w}}$. Time is shown in generations. The parameters are $b = c = 1$, $f = 0.18$, $\rho_a = 0.74$, $\rho_s = 0.76$, $\mu = 10^{-9}$ (left panels) and $\mu = 10^{-7}$ (right panels). The initial allele frequencies are $m^0 = 0.99$, $p^0 = q^0 = 0.9999$, and all initial linkage disequilibria are zero. Thus, the initial conditions are very close to the stable limit cycle that exists when M_1 is fixed. The main effect of the higher mutation rate is that the replacement occurs on a much longer time scale.

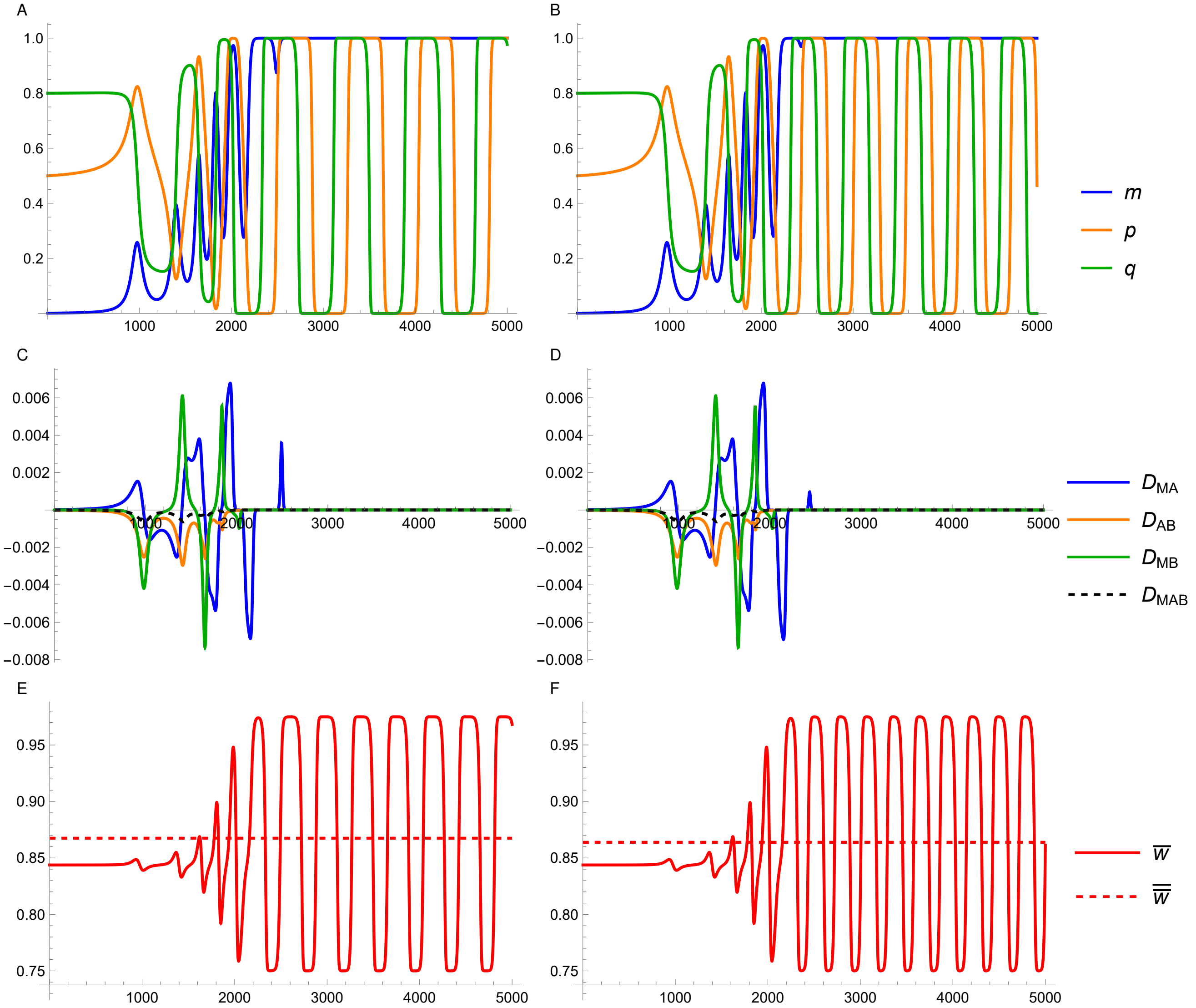

Fig. S3. Invasion of a rare specific modifier allele M_1 and eventual replacement of the resident specific allele M_0. This leads to the exclusive use of PRDM9 hotspots. Panels A and B show the trajectories of the allele frequencies m, p, and q of M_1, A_1, and B_1. Panels C and D show the four linkage disequilibria. Panels E and F show the time-dependent mean fitness $\bar{w}$ and the mean fitness averaged over all generations, $\bar{\bar{w}}$. Time is shown in generations. The parameters are $b = c = 1$, $f = 0.25$, $\rho_a = 0.6$, $\rho_s = 0.9$, $\mu = 10^{-9}$ (left panels) and $\mu = 10^{-7}$ (right panels). The initial allele frequencies are $m^0 = 0.001$, $p^0 = 0.5$, $q^0 = 0.8$, and all initial linkage disequilibria are zero. Thus, the initial conditions are very close to the stable limit cycle that exists when M_1 is fixed. For such a relatively strong advantage caused by symmetric binding, the effect of the higher mutation rate is very weak: it reduces the waiting time near hotspots and coldspots for the next transition.

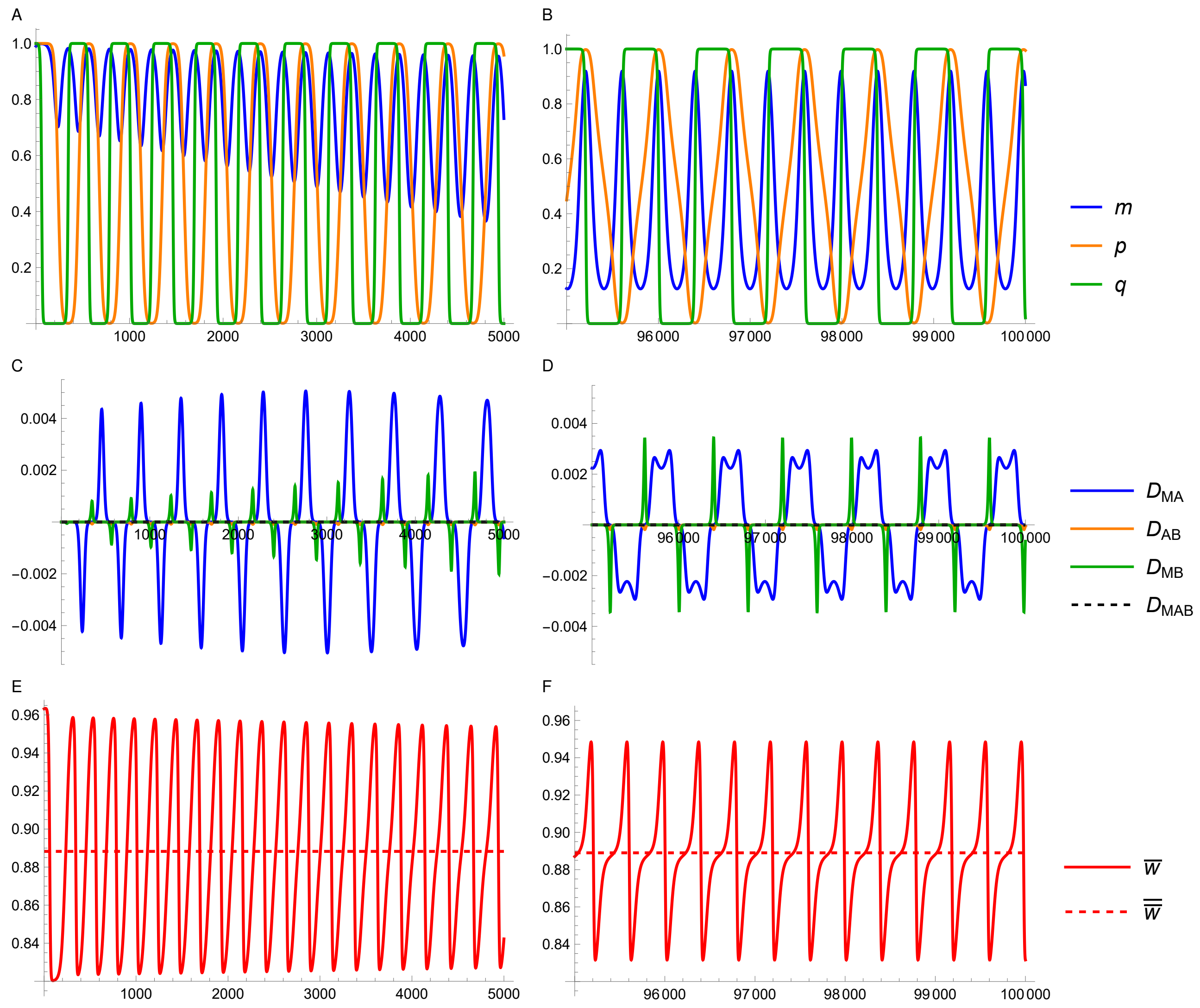

Fig. S4. Invasion of the unspecific modifier allele M_0 and eventual oscillatory coexistence of M_0 and M_1 (as well as of A_1 and A_2, and of B_1 and B_2). This leads to oscillatory use of PRDM9 and non-PRDM9 hotspots. Panels A and B show the trajectories of the allele frequencies m, p, and q of M_1, A_1, and B_1. The amplitude of m increases until about generation $25\,000$ when it begins to stabilize. Panels C and D show the four linkage disequilibria. Time is shown in generations. The parameters are $b = c = 1$, $f = 0.18$, $\rho_a = 0.7$, $\rho_s = 0.8$, and $\mu = 10^{-7}$. The initial allele frequencies are $m^0 = 0.99$, $p^0 = q^0 = 0.9999$ (initially LD is absent). The corresponding dynamics for $\mu = 10^{-9}$ are not shown because they are very similar to those in the left panels of Fig. S2. In fact, Fig. 4 in the main text suggests that in this case M_0 always replaces M_1.

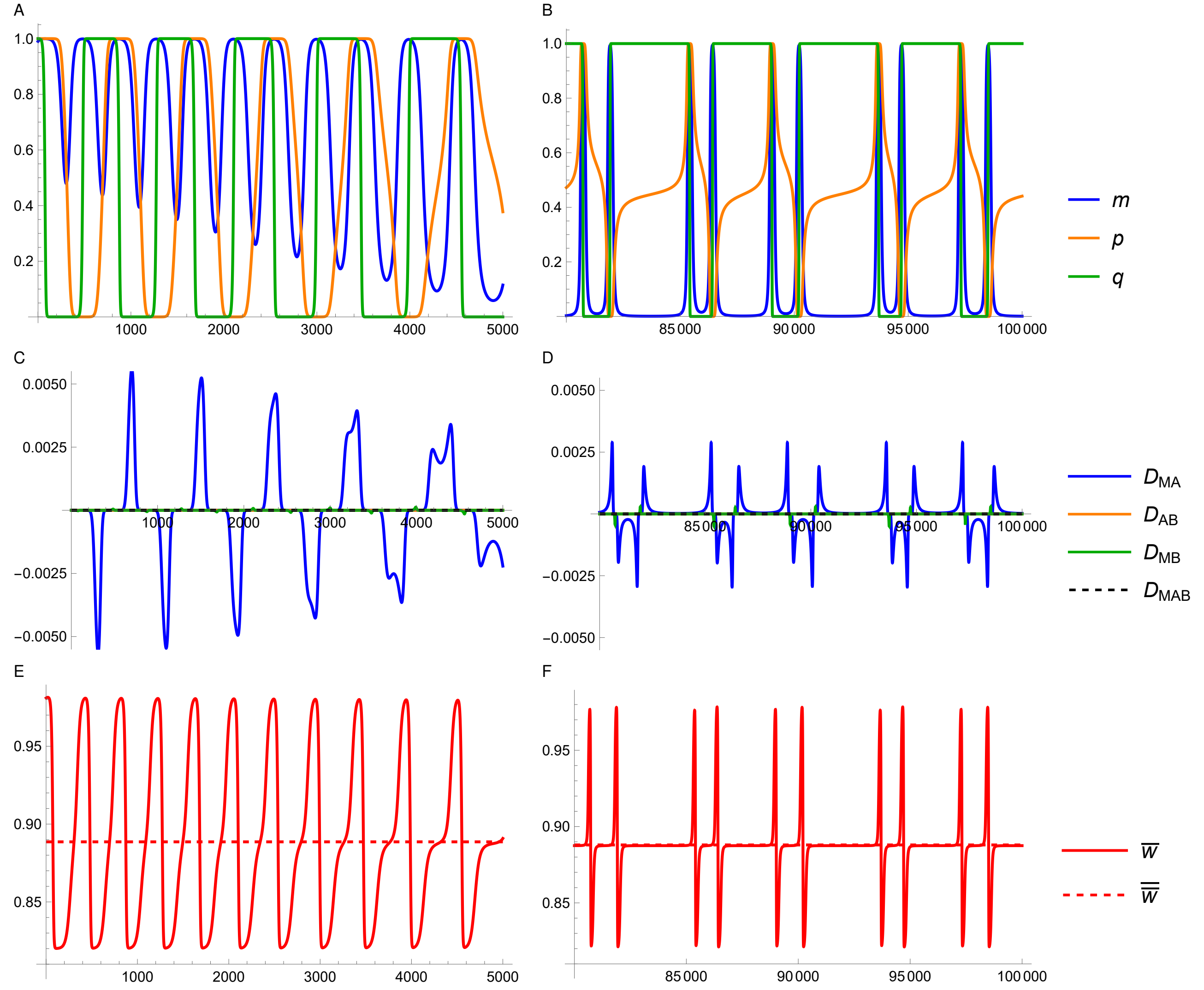

Fig. S5. Invasion of the unspecific modifier allele M_0 and eventual coexistence of M_0 and M_1 by complicated oscillatory dynamics of all alleles. Panels A and B show the trajectories of the allele frequencies m, p, and q of M_1, A_1, and B_1. The amplitude of m increases in a cyclical manner until about generation $6\,000$; then the complicated dynamics with a period of at least 4 becomes established. Panels C and D show the four linkage disequilibria. The parameters are $b = c = 1$, $f = 0.18$, $\rho_a = 0.6$, $\rho_s = 0.9$, and $\mu = 10^{-9}$. The initial allele frequencies are $m^0 = 0.99$, $p^0 = q^0 = 0.9999$ (initially LD is absent). For $\mu = 10^{-7}$ and the same choice of b, c, f, ρ_a, and ρ_s, M_0 can invade initially but is lost in the long run. The corresponding dynamics is shown in the left panels of Fig. S6.

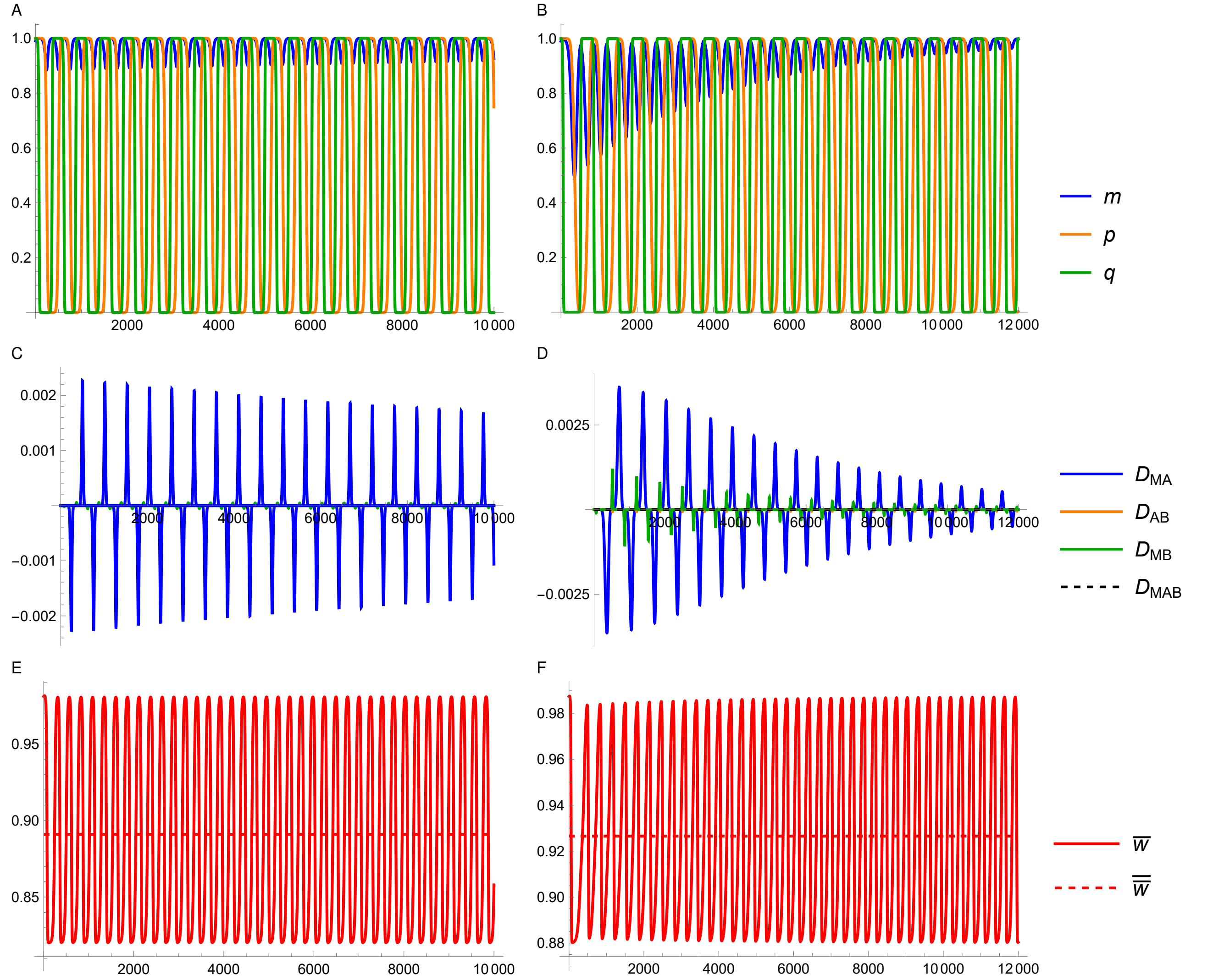

Fig. S6. Invasion of the unspecific modifier allele M_0 but eventual loss of M_0. Panels A and B show the trajectories of the allele frequencies m, p, and q of M_1, A_1, and B_1. Panels C and D show the four linkage disequilibria. In all panels the parameters are $b = c = 1$, $\rho_a = 0.6$, $\rho_s = 0.9$. In the left panels, we have $f = 0.18$ and $\mu = 10^{-7}$, in the right panels we have $f = 0.12$ and $\mu = 10^{-9}$. The initial allele frequencies are $m^0 = 0.99$ and $p^0 = q^0 = 0.9999$ in both panels (initially LD is absent everywhere). In each case, the unspecific modifier M_0 can invade initially, but it is eliminated in the long run. For the data in A it takes nearly 500 000 generations until M_0 has decayed below 10^{-6}, for those in B, it takes about 60 000 generations (results not shown). These and other figures and additional numerical results suggest that higher mutation rates tend to have a positive effect on cycling and resistance of the specific modifier against invasion of unspecific modifiers.

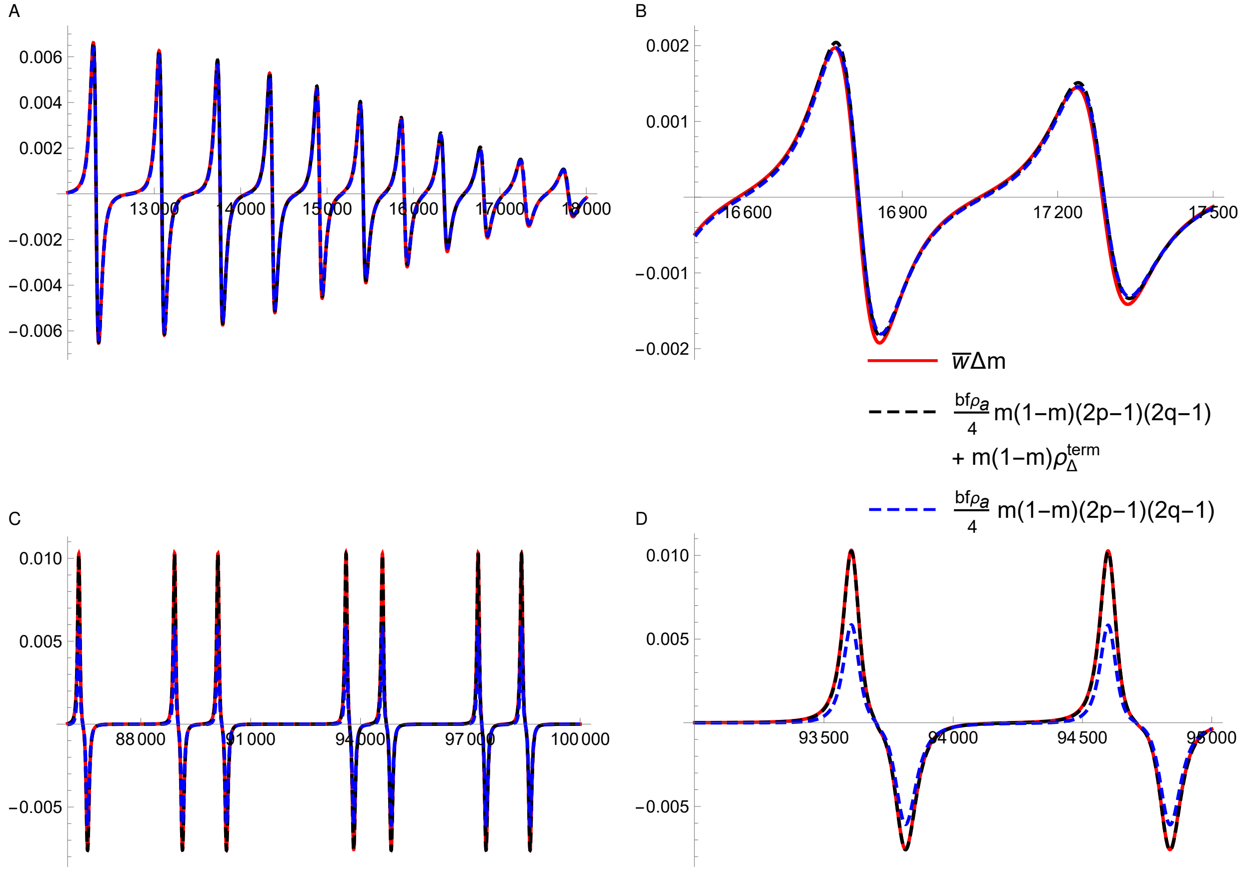

Fig. S7. This figure shows the scaled per-generation response $\bar{w}\Delta m$ of the modifier allele M_1, the approximation given in $\mathrm{Eq.}$ (32) (with D_{AB} set to 0), as well as $\frac{bf\rho_a}{4}m(1-m)(1-2p)(1-2q)$. Panels A and B show data corresponding to the right panels in Fig. S2 ($f = 0.18$, $\rho_a = 0.74$, $\rho_s = 0.76$, $\mu = 10^{-7}$), whereas C and D show data corresponding to Fig. S5 ($f = 0.18$, $\rho_a = 0.6$, $\rho_s = 0.9$, $\mu = 10^{-9}$). In each case only a relatively small number of generations is shown in order to improve the visibility of the effects. The panels on the right-hand side are zoomed-in versions of the left side. Panel B shows that the approximation $\mathrm{Eq.}$ (32) (black dashed curve) differs slightly from the true values (red curve). Together with panel D in Fig. S2, which shows that for an intermediate range of generations the linkage disequilibria D_{AB} and D_{MB} are relatively large, this indicates that the neglect of linkage disequilibria is the cause of this deviation. Panels C and D show that neglecting the fitness advantage of symmetric binding (blue dashed curve) leads to a poor approximation because $\rho_s - \rho_a = 0.3$ is relatively large. Here, linkage disequilibria play essentially no role. In the legend we use the abbreviation $\rho_\Delta^{\text{term}} = \frac{b^2 f}{4}(\rho_s - \rho_a)(2q-1)\Big[(2p-1) + \frac{1}{2}\Big((1-p)^2 + p^2\Big)(2q-1)\Big]$ for the sum of the terms in $\mathrm{Eq.}$ (32) that depend on $(\rho_s - \rho_a)$ but not on D_{AB}.

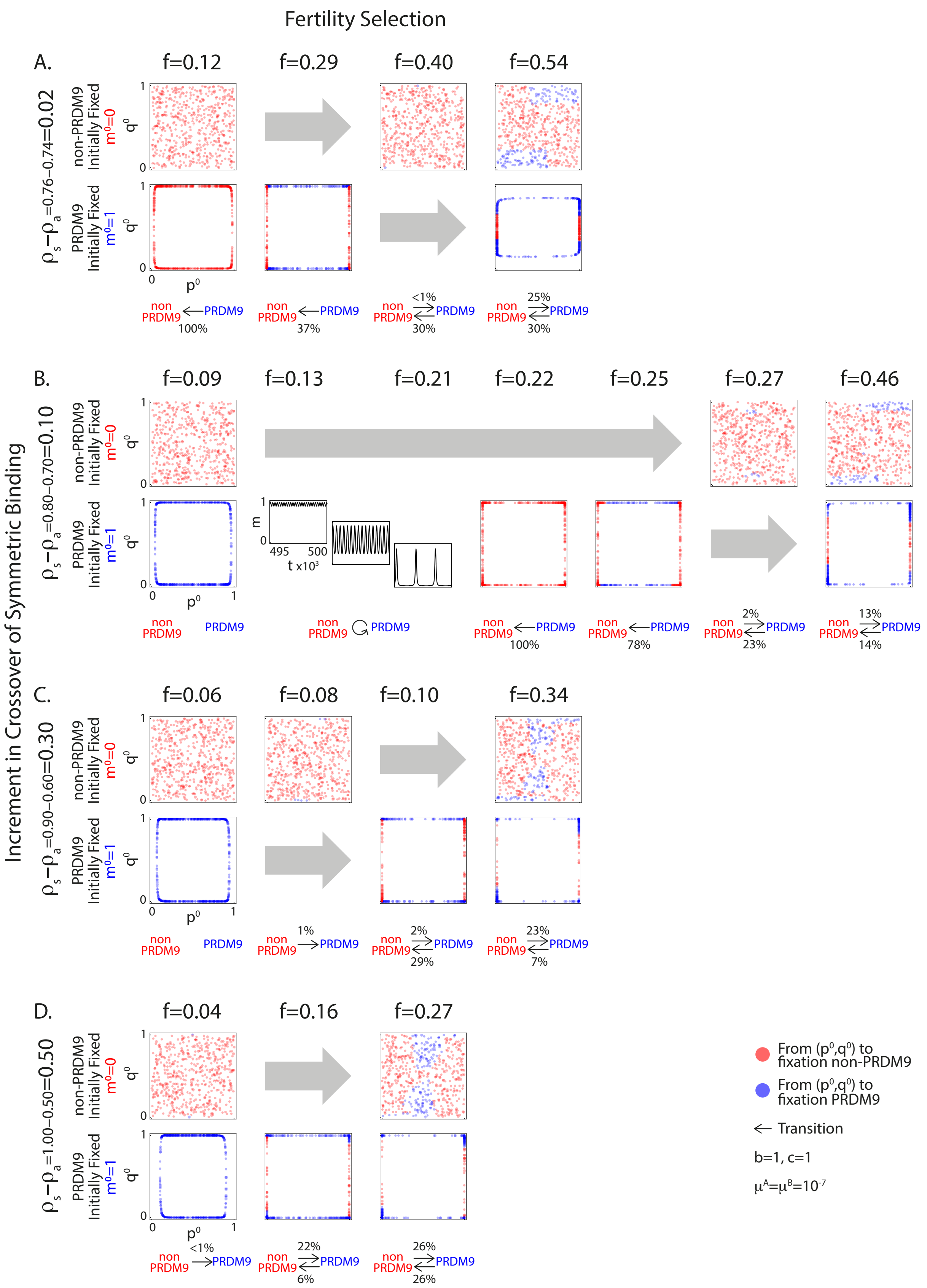

Fig. S8. Transitions between types of recombination hotspots when symmetric binding is advantageous. This figure is analogous to Fig. 6 in the main text except that here the mutation rate at targeting and target loci is $\mu = 10^{-7}$ instead of 10^{-9}.

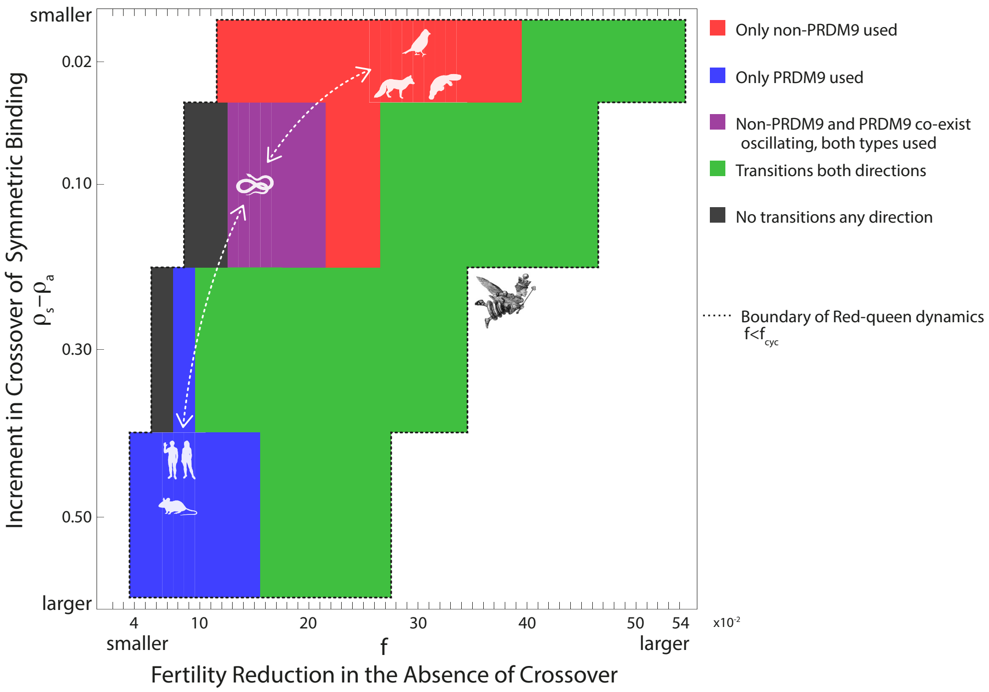

Fig. S9. Types of replacement behaviour in different regions of the parameter space. This figure is analogous to Fig. 7 in the main text except that here the mutation rate at targeting and target loci is $\mu = 10^{-7}$ instead of 10^{-9}. We note that we found stable oscillations for $\rho_s - \rho_a = 0.9 - 0.6$ and $f = 0.175$, but neither for $f \leq 0.17$ nor $f \geq 0.18$. This region (purple) is not shown in the graph because to generate the graph we used increments in f of 0.02.

References

1. F Úbeda, T Russell, V Jansen, PRDM9 and the evolution of recombination hotspots. *Theor. Popul. Biol.* **126**, 19 –32 (2019).
2. F Úbeda, F Fyon, R Bürger, The recombination hotspot paradox: Co-evolution between PRDM9 and its target sites. *Theor. Popul. Biol.* **153**, 69–90 (2023).
3. R Bürger, *The mathematical theory of selection, recombination, and mutation.* (John Wiley & Sons), (2000).
4. S Wright, The distribution of gene frequencies in populations. *Proc. Natl. Acad. Sci.* **23**, 307–320 (1937).
5. NH Barton, M Turelli, Natural and sexual selection on many loci. *Genetics* **127**, 229–255 (1991).
6. S Myers, L Bottolo, C Freeman, G McVean, P Donnelly, A fine-scale map of recombination rates and hotspots across the human genome. *Science* **310**, 321–324 (2005).
7. N Arnheim, P Calabrese, I Tiemann-Boege, Mammalian meiotic recombination hot spots. *Annu. Rev. Genet.* **41**, 369–399 (2007).
8. K Paigen, P Petkov, Mammalian recombination hot spots: properties, control and evolution. *Nat. Rev. Genet.* **11**, 221 (2010).
9. F Baudat, Y Imai, B De Massy, Meiotic recombination in mammals: localization and regulation. *Nat. Rev. Genet.* **14**, 794 (2013).
10. PL Oliver, et al., Accelerated evolution of the prdm9 speciation gene across diverse metazoan taxa. *PLoS Genet.* **5** (2009).
11. F Baudat, et al., PRDM9 is a major determinant of meiotic recombination hotspots in humans and mice. *Science* **327**, 836–840 (2010).
12. F Úbeda, JF Wilkins, The red queen theory of recombination hotspots. *J. evolutionary biology* **24**, 541–553 (2011).
